# Assessment of Type I Interferon Signaling in Pediatric Inflammatory Disease

**DOI:** 10.1007/s10875-016-0359-1

**Published:** 2016-12-09

**Authors:** Gillian I. Rice, Isabelle Melki, Marie-Louise Frémond, Tracy A. Briggs, Mathieu P. Rodero, Naoki Kitabayashi, Anthony Oojageer, Brigitte Bader-Meunier, Alexandre Belot, Christine Bodemer, Pierre Quartier, Yanick J. Crow

**Affiliations:** 1 Faculty of Biology, Medicine and Health, School of Biological Sciences, Division of Evolution and Genomic Sciences, University of Manchester, Manchester, UK; 2 INSERM UMR 1163, Laboratory of Neurogenetics and Neuroinflammation, Paris, France; 3 Sorbonne-Paris-Cité, Institut Imagine, Hôpital Necker Enfants Malades, Assistance Publique-Hôpitaux de Paris, Paris Descartes University, Paris, France; 4 General Pediatrics, Infectious Disease and Internal Medicine Department, Hôpital Robert Debré, AP-HP, Paris, France; 5 Pediatric Hematology-Immunology and Rheumatology Department, Hôpital Necker Enfants Malades, Assistance Publique-Hôpitaux de Paris, Paris, France; 6 Manchester Centre for Genomic Medicine, St Mary’s Hospital, Manchester Academic Health Science Centre, Central Manchester University Hospitals NHS Foundation Trust, Manchester, UK; 7 INSERM UMR 1163, Laboratory of Immunogenetics of Pediatric Autoimmunity, Paris, France; 8 Pediatric Rheumatology, Nephrology and Dermatology Department, Hospices Civils de Lyon, Lyon, France; 9 CIRI, Centre International de Recherche en Infectiologie, INSERM, U1111, CNRS UMR5308, Ecole Normale Supérieure de Lyon, Université Lyon 1, Lyon, France; 10 Department of Paediatric Dermatology, Reference Centre for Rare Skin Disorders (MAGEC), Hôpital Necker Enfants Malades, Assistance Publique-Hôpitaux de Paris, Paris, France; 11 Laboratory of Neurogenetics and Neuroinflammation, Institut Imagine, 3rd Floor, Room 309, 24 Boulevard du Montparnasse, 75015 Paris, France

**Keywords:** Interferon, interferonopathy, autoinflammation, autoinflammatory disease

## Abstract

**Purpose:**

Increased type I interferon is considered relevant to the pathology of a number of monogenic and complex disorders spanning pediatric rheumatology, neurology, and dermatology. However, no test exists in routine clinical practice to identify enhanced interferon signaling, thus limiting the ability to diagnose and monitor treatment of these diseases. Here, we set out to investigate the use of an assay measuring the expression of a panel of interferon-stimulated genes (ISGs) in children affected by a range of inflammatory diseases.

**Design, Setting, and Participants:**

A cohort study was conducted between 2011 and 2016 at the University of Manchester, UK, and the Institut Imagine, Paris, France. RNA PAXgene blood samples and clinical data were collected from controls and symptomatic patients with a genetically confirmed or clinically well-defined inflammatory phenotype. The expression of six ISGs was measured by quantitative polymerase chain reaction, and the median fold change was used to calculate an interferon score (IS) for each subject compared to a previously derived panel of 29 controls (where +2 SD of the control data, an IS of >2.466, is considered as abnormal). Results were correlated with genetic and clinical data.

**Results:**

Nine hundred ninety-two samples were analyzed from 630 individuals comprising symptomatic patients across 24 inflammatory genotypes/phenotypes, unaffected heterozygous carriers, and controls. A consistent upregulation of ISG expression was seen in 13 monogenic conditions (455 samples, 265 patients; median IS 10.73, interquartile range (IQR) 5.90–18.41), juvenile systemic lupus erythematosus (78 samples, 55 patients; median IS 10.60, IQR 3.99–17.27), and juvenile dermatomyositis (101 samples, 59 patients; median IS 9.02, IQR 2.51–21.73) compared to controls (78 samples, 65 subjects; median IS 0.688, IQR 0.427–1.196), heterozygous mutation carriers (89 samples, 76 subjects; median IS 0.862, IQR 0.493–1.942), and individuals with non-molecularly defined autoinflammation (89 samples, 69 patients; median IS 1.07, IQR 0.491–3.74).

**Conclusions and Relevance:**

An assessment of six ISGs can be used to define a spectrum of inflammatory diseases related to enhanced type I interferon signaling. If future studies demonstrate that the IS is a reactive biomarker, this measure may prove useful both in the diagnosis and the assessment of treatment efficacy.

**Electronic supplementary material:**

The online version of this article (doi:10.1007/s10875-016-0359-1) contains supplementary material, which is available to authorized users.

## Introduction

Given their potent and broad effects, the type I interferons represent both key molecules in anti-viral defense and potential mediators of inflammatory disease. As such, the induction, transmission, and resolution of the interferon response are tightly regulated. Mendelian disorders associated with a persistent upregulation of type I interferons, the so-called type I interferonopathies, and related non-monogenic phenotypes, most particularly systemic lupus erythematosus (SLE) and dermatomyositis (DM), represent examples of a disturbance of the homeostatic control of this complex system [1–4]. The recognition of these disorders will become of increasing clinical importance as “anti-interferon” treatments are developed [5].

Surprisingly, no routine laboratory test exists in current medical practice for the assessment of type I interferon signaling. Although a cytopathic protection assay, measuring anti-viral activity in patient material, was central in defining the first described monogenic type I interferonopathy, Aicardi-Goutières syndrome (AGS), this assay is neither widely available nor easily automated [6, 7]. Furthermore, type I interferon mRNA and protein assays in peripheral blood mononuclear cells (PBMCs) have proven insensitive as disease biomarkers, leading to the development of a variety of proxy assays [8–12]. Such low levels of circulating type I interferons presumably reflect their high biological potency, with most cells expressing a type I interferon receptor.

Based on the initial work of others on SLE [13, 14], we previously defined the characteristics of a test involving quantitative PCR (qPCR) assessment of six interferon-stimulated genes (ISGs) using RNA extracted from PBMCs of patients with AGS [15]. Here, we now report the use of this interferon signature in a large cohort of patients and controls screened for type I interferon signaling status. These data allow for a better understanding of the practical application, interpretation, and utility of such an assay, as well as an improved characterization of the relationship of distinct diseases to type I interferon and the core clinical features that should alert a physician to the possibility of a type I interferon-related disorder.

## Methods

### Patient Cohort

We tested patients, and in certain cases parents and siblings to these patients, referred to us for assessment of type I interferon status. Clinical and molecular data were evaluated through direct contact and/or collected via collaborating physicians. We included cases with molecularly confirmed monogenic inflammatory diseases and a number of patients with non-molecularly defined clinical phenotypes which were either known to be, or we hypothesized might be, associated with increased type I interferon signaling. Control samples comprised an ethnically diverse group of individuals who self-reported not to have any medical condition. We also included in our control group parents or siblings to a person with an autosomal dominant interferonopathy where the parent/sibling was negative for the familial mutation. Neither patients nor controls demonstrated features of infection at the time of sampling.

### Interferon Score (IS)

Blood was collected into PAXgene tubes (PreAnalytix) and, after being kept at room temperature for between 1 and 72 h, was frozen at −20 °C until extraction. Total RNA was extracted from whole blood using a PAXgene (PreAnalytix) RNA isolation kit. RNA concentration was assessed using a spectrophotometer (FLUOstar Omega, Labtech). Quantitative reverse transcription polymerase chain reaction (qPCR) analysis was performed using the TaqMan Universal PCR Master Mix (Applied Biosystems) and cDNA derived from 40 ng total RNA. Using TaqMan probes for *IFI27* (Hs01086370_m1), *IFI44L* (Hs00199115_m1), *IFIT1* (Hs00356631_g1), *ISG15* (Hs00192713_m1), *RSAD2* (Hs01057264_m1), and *SIGLEC1* (Hs00988063_m1), the relative abundance of each target transcript was normalized to the expression level of *HPRT1* (Hs03929096_g1) and *18S* (Hs999999001_s1) and assessed with the Applied Biosystems StepOne Software v2.1 and DataAssist Software v.3.01. For each of the six probes, individual (patient and control) data were expressed relative to a single calibrator (control C25) (Table S1). The median fold change of the six ISGs, when compared to the median of previously collected 29 healthy controls, was used to create an IS for each individual. Relative quantification (RQ) is equal to 2^−ΔΔCt^, i.e., the normalized fold change relative to the control data. In this way, we define an abnormal IS as being greater than +2 standard deviations above the mean of this control group, i.e., 2.466.

### Statistics

In the absence of a normal distribution, ISG levels and ISs were log-transformed and analyzed with parametric testing (*t* tests for two groups, one-way ANOVA for more than two groups). Tests for multiple comparisons, Bonferroni’s multiple comparison test or Dunnett’s multiple comparison test as appropriate, were applied as detailed in the figure legends. GraphPad Prism version 6 for Mac OS X was used for statistical analysis.

### Ethics Approvals

The study was approved by the Leeds (East) Research Ethics Committee (reference number 10/H1307/132) and by the Comité de Protection des Personnes (ID-RCB/EUDRACT: 2014-A01017-40).

## Results

### Overall Cohort Characteristics

**Fig. 1:**
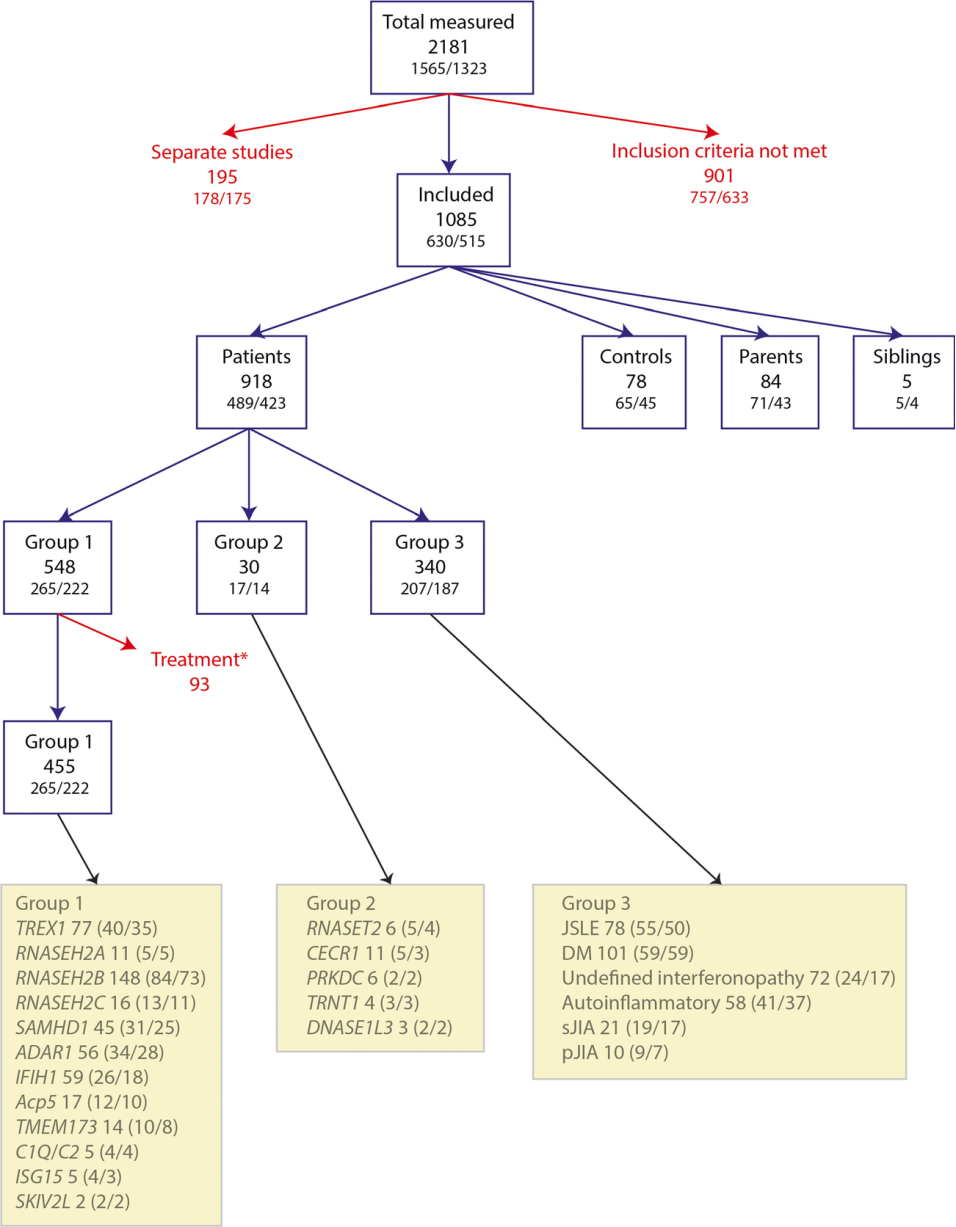
Flow chart showing inclusion/exclusion criteria for study participation. The number of measurements (samples) is given, together with the number of individuals/number of families below/in brackets. Treatment* refers to samples excluded (*n* = 93) because patients were on reverse transcriptase or JAK inhibitors

Over a period of 6 years, we tested a total of 2181 samples for an interferon signature. These samples were from 1565 individuals, comprising 75 persons considered as controls (96 samples); 1264 patients (1827 samples); and 209 parents (241 samples) and 17 siblings (17 samples) of affected patients (Fig. 1).

We excluded the following from this total of 2181 samples: 187 samples from controls, parents, and siblings where data on age, sex, or genotype were unavailable or where a proband was also excluded (see below); 42 samples from 12 AGS patients under treatment with reverse transcriptase inhibitors (https://clinicaltrials.gov/ct2/show/NCT02363452?term=Aicardi+Goutieres&rank=1); 51 samples from seven *IFIH1*- and *TMEM173*-mutated patients under treatment with the JAK1/2 inhibitor ruxolitinib [5]; 175, seven, and 13 results from 161, seven, and 10 individuals with, respectively, adult-onset SLE/mixed connective tissue disease, retinal vasculopathy and cerebral leukodystrophy (RVCL), and *STAT1* gain-of-function mutations which were performed as part of separate studies. We also excluded 714 samples from 597 patients where there was no genetic diagnosis and/or where phenotypic data were limited/allowed for no definite clinical diagnosis, there was no family history of similarly affected relatives, and for whom we had tested less than three ISs.

Following the above exclusions, our cohort consisted of 992 samples from 630 individuals, specifically: 78 samples from 65 controls (including 31 parents and seven siblings to patients with a molecularly proven autosomal dominant type I interferonopathy, most particularly due to heterozygous mutations in *TREX1*, *ADAR1*, *IFIH1*, and *TMEM173*, where the mutation was shown to have occurred de novo) (Table S1); 84 samples from 71 parents and five samples from five siblings where the parent/sibling of a person with biallelic mutations in a known type I interferonopathy-causing gene was shown to be heterozygous for one familial mutation (Table S2); and 825 samples from 489 patients which we divided into three groups for ease of analysis (Table 1). Group 1 comprises patients with one or more interferon signature reading(s) and a confirmed molecular diagnosis in any of the following 13 genes considered to have a proven link to type I interferon production/signaling: *TREX1* (Table S3), *RNASEH2A* (Table S4), *RNASEH2B* (Table S5), *RNASEH2C* (Table S6), *SAMHD1* (Table S7), *ADAR1* (Table S8), *IFIH1* (Table S9), *ACP5* (Table S10), *TMEM173* (Table S11), *C1Q* (Table S12), *C2* (Table S12), *ISG15* (Table S13), and *SKIV2L* (Table S14). Of note, we only included patients with biallelic mutations in these genes, except for those individuals with recognized dominant mutations in *TREX1* (at positions p.Asp18 and p.Asp200), *ADAR1* (at position p.Gly1007), *IFIH1*, and *TMEM173*. In group 2, we included patients with a confirmed molecular diagnosis of any other genotype, where at least one patient from each of at least two different families tested positive for an interferon signature on at least one occasion. This group thus comprised patients with mutations in *DNASE1L3* (Table S15), *PRKDC* (Table S16), *CECR1* (Table S17), *RNASET2* (Table S18), and *TRNT1* (Table S19). Finally, in group 3, we included patients with a clinical diagnosis of juvenile SLE (JSLE) (Table S20); juvenile DM (JDM) (Table S21); systemic juvenile idiopathic arthritis (sJIA) (Table S24) and other, non-systemic, JIA (pJIA) (Table S25); a molecularly undetermined phenotype which was clinically labeled as autoinflammatory (defined here as unexplained recurrent fevers and/or organ-specific features with elevated markers of systemic inflammation in the absence of autoimmunity and underlying infection) (Table S23); and patients with a phenotype variably comprising neurological, dermatological, rheumatological, and immunological features reminiscent of the known type I interferonopathy spectrum (Table S22), who screened negative for mutations in relevant type I interferonopathy-associated genes and who demonstrated a positive IS on three or more occasions or had a similarly affected relative with a minimum of three positive ISs shared between affected family members.

**Table 1:** Summary data of median (and interquartile range, IQR) of interferon score in patients categorized by genotype/phenotype within each patient group

	Number of samples/patients	Number (percentage) with positive interferon score^a^	Median (IQR)
Controls	78/65	7 (8.97%)	0.688 (0.427–1.196)
Group 1 patients combined	455/265	412 (90.55%)	10.73 (5.889–18.41)
*TREX1*	77/40	74 (96.10%)	10.89 (5.859–19.79)
*RNASEH2A*	11/5	11 (100%)	8.303 (4.215–19.59)
*RNASEH2B*	148/84	115 (77.70%)	6.101 (2.744–10.84)
*RNASEH2B* with positive score	115		7.731 (5.387–11.76)
*RNASEH2C*	16/13	16 (100%)	9.104 (6.819–13.78)
*SAMHD1*	45/31	45 (100%)	12.51 (9.215–16.53)
*ADAR1*	56/34	52 (92.86%)	16.16 (8.801–25.39)
*IFIH1*	59/26	59 (100%)	16.12 (11.15–22.03)
*ACP5*	17/12	14 (82.35%)	21.43 (3.264–32.26)
*TMEM173*	14/10	14 (100%)	16.05 (9.541–26.20)
*C1Q*	4/3	4 (100%)	16.86 (4.374–39.41)
*C2*	1/1	1 (100%)	11.90
*ISG15*	5/4	5 (100%)	21.24 (13.22–31.77)
*SKIV2L*	2/2	2 (100%)	24.67 (22.04–27.31)
Parents + siblings of group 1 patients	89/76	19 (21.35%)	0.862 (0.493–1.942)
Group 2 patients combined	30/17	23 (76.67%)	5.728 (2.604–9.805)
*CECR1*	11/5	9 (81.82%)	5.653 (3.349–7.053)
*RNASET2*	6/5	5 (83.33%)	4.288 (2.512–6.338)
*PRKDC*	6/2	5 (83.33%)	17.06 (4.582–22.29)
*TRNT1*	4/3	2 (50%)	4.048 (1.324–7.702)
*DNASE1L3*	3/2	2 (66.67%)	10.83 (0.374–11.00)
Group 3 patients combined	340/207	235 (69.12%)	
JSLE	78/55	64 (82.05%)	10.60 (3.986–17.27)
JDM	101/59	76 (75.25%)	9.019 (2.507–21.73)
Molecularly undefined interferonopathy	72/24	67 (93.06%)	9.385 (6.675–13.98)
sJIA	21/19	6 (28.57%)	0.676 (0.496–6.421)
pJIA	10/9	0 (0%)	0.800 (0.387–1.074)
Autoinflammatory	58/41	22 (37.93%)	1.252 (0.590–4.064)

*JSLE* juvenile systemic lupus erythematosus, *JDM* juvenile DM, *sJIA* systemic juvenile idiopathic arthritis (JIA), *pJIA* non-systemic JIA

^a^Positive interferon score ≥2.466

### Results in Controls

Of 78 ISs from 65 controls, seven (9.0%) were abnormal (median IS 0.688, IQR 0.427–1.196) (Table 1, Table S1). In only two of these was the IS above five. The IS was measured on more than one occasion in 12 control individuals, with three persons being sampled three times. On 26 of 27 occasions, the IS was normal (Figure S1), the one positive sample returning an IS of 23.4 which was normal on repeat sampling. Controls ranged from 1 year to 93 years of age and there was no correlation between age and IS (*p* = NS). Thirty-five of 65 controls were female.

### Results in Group 1

**Fig. 2:**
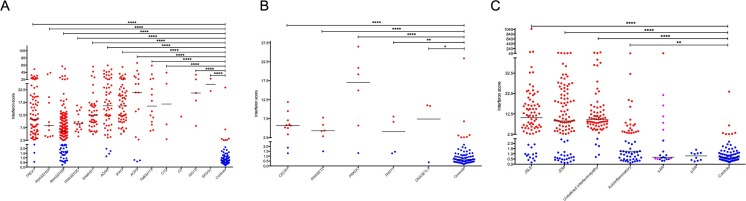
Interferon score plotted for each sample according to genotype/phenotype. **a** 455 group 1 patient samples. **b** 30 group 2 patient samples. **c** 340 group 3 patient samples. *Black horizontal lines* represent the median for each patient group. Interferon scores calculated from the median fold change in RQ (relative quantification) values of a panel of six interferon-stimulated genes (ISGs). *Blue dots* represent an interferon score of less than 2.466. *Red dots* represent an interferon score of greater than 2.466. *Magenta dots* represent patients treated with IL1 blockade. Analyzed by one-way ANOVA with Dunnett’s multiple comparison test

**Fig. 3:**
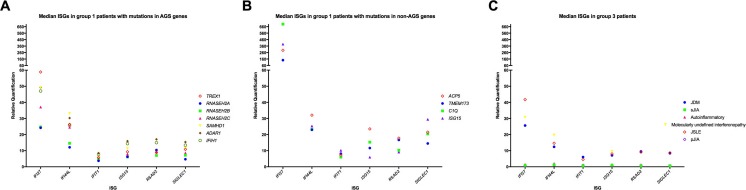
Median fold expression of six interferon-stimulated genes according to genotype. Median relative quantification (RQ) value for each of six interferon-stimulated genes (ISGs) measured in **a** 74 *TREX1*, 11 *RNASEH2A*, 115 *RNASEH2B*, 16 *RNASEH2C*, 45 *SAMHD1*, 52 *ADAR1*, and 59 *IFIH1* samples with a positive (>2.466) interferon score; **b** 14 *ACP5*, 14 *TMEM173*, four *C1QA*, and five *ISG15* samples with a positive (>2.466) interferon score; **c** 101 JDM, 21 sJIA, 58 autoinflammatory, 72 molecularly undefined interferonopathy, 78 JSLE, and ten pJIA. RQ is equal to 2^−∆∆Ct^, i.e., the normalized fold change relative to a control

In this group, we measured 455 interferon signatures from 265 mutation-positive patients belonging to 222 families. All of these genotypes were associated with a significant upregulation of type I interferon signaling (median IS 10.73, IQR 5.90–18.41) (Table 1, Fig. 2a, Figure S2) compared to controls. Thirty-three of 148 samples (22.30%) from patients with mutations in *RNASEH2B* demonstrated a normal IS, and a lower median IS (6.10, IQR 2.74–10.84) was observed in this group compared to all other genotypes (median IS 13.14, IQR 8.12–21.54), with the next lowest median ISs associated with mutations in the two other proteins comprising the RNase H2 complex. A comparison of the median RQ value for each of the six individual ISGs across the genotypes comprising group 1 revealed higher fold induction of *IFI27* and *SIGLEC1* in patients mutated in *ACP5*, *TMEM173*, *C1Q*, and *ISG15* compared to all other group 1 genotypes and to patients in group 3 (Fig. 3).

Ten samples from patients mutated in *TREX1* (two of 40 patients; three of 77 samples), *ADAR1* (four of 34 individuals; four of 56 samples), and *ACP5* (two of 12 patients; three of 17 samples) also returned normal results (Fig. 2a, Table S26), versus none of 95 patients (none of 157 samples) with the nine other genotypes. We note that some, but not all, of these interferon signature-negative patients demonstrated a milder clinical phenotype in comparison with other cases mutated in these same genes (data not shown). Of further note, one of the two patients with *ACP5*-related disease with a normal IS was on high-dose immunosuppressants and was reported to have responded well clinically at the time of sampling.

### Results in Group 2

In this group, we measured 30 interferon signatures from 17 mutation-positive patients belonging to 14 families (Fig. 2b, Figure S3). For each of the five genotypes, *CECR1* (11 samples, five patients), *RNASET2* (six samples, five patients), *PRKDC* (six samples, two patients), *TRNT1* (four samples, three patients), and *DNASE1L3* (three samples, two patients), we recorded positive ISs in at least one patient from each of at least two different families (Fig. 2b).

### Results in Group 3

Group 3 includes patients with molecularly undefined phenotypes, where we measured 340 interferon signatures from 207 patients belonging to 187 families (Fig. 2c, Figure S4). Our data mirror the results from multiple studies demonstrating an upregulation of interferon signaling in a significant proportion of individuals with JSLE and JDM, where 82% (64 of 78) and 75% (76 of 101) of samples were abnormal in these two phenotypic groupings, respectively (JSLE: median IS 10.60, IQR 3.99–17.27; JDM: median IS 9.02, IQR 2.51–21.73) (Table 1). We note that a number of these patients were under treatment, and some had clinically quiescent disease at the time of sampling. In contrast, a lower proportion (14 of 41) of patients with a clinical diagnosis of a non-molecularly determined autoinflammatory phenotype returned a positive IS (median 1.25, IQR 0.59–4.06). Considering another complex disease, sJIA, we identified five of 19 patients with a positive IS, 11 of whom were being treated with interleukin 1 (IL1) blockade. Finally, we also included in this group 72 samples from 24 patients belonging to 17 families who did not carry a mutation in known clinically relevant type I interferonopathy-associated genes and where we recorded an upregulation of type I interferon signaling measured on at least three occasions—thus, likely indicative of a true association with enhanced type I interferon signaling (median IS 9.38, IQR 6.67–13.98).

### Results in Parents and Siblings of Patients in Group 1

The large majority of samples from proven heterozygous carrier parents (66 of 84 samples) and siblings (four of five samples) to patients with biallelic mutations in *AGS1-7* and *ACP5* did not demonstrate an interferon signature (median IS 0.862, IQR 0.493–1.942) (Figure S5). Ten of the 18 abnormal parental signatures were recorded in individuals heterozygous for a mutation in *ADAR1*.

## Discussion

We present an overview of our experience of screening a large cohort of patients and controls for an induction of type I interferon signaling in whole blood by quantifying the expression of six ISGs—*IFI27*, *IFI44L*, *IFIT1*, *ISG15*, *RSAD2*, and *SIGLEC1*. There is no consensus as to the precise set of genes to measure when testing for an interferon signature. Nor is there a universally accepted method for calculating an IS based on a composite of multi-gene transcript upregulation. Prior to this study, we measured the expression of 15 ISGs in patients with mutations in *ADAR1* [16]. Based on those results, and a series of unpublished genome-wide expression experiments, we then focused on six ISGs that were highly expressed in individuals from a cohort of molecularly defined AGS patients [15]. The median fold change of the six ISGs compared to the median of 29 healthy controls was used to create an IS for each patient, with a value greater than two standard deviations above the mean score of the controls (>2.466) being designated as positive. In this previously published work, we also showed that our IS positively correlated with an assay of anti-viral cytopathic protection.

The extended control data set presented here confirms that the large majority of healthy persons do not demonstrate an upregulation of type I interferon signaling, irrespective of age or sex. In contrast, work published by many groups has shown that enhanced type I interferon signaling is a reliable biomarker of a number of clinical phenotypes [13, 14, 17]. Given that our data recapitulate the results of these genome-wide expression studies, we consider that the simple screening assay presented here has validity as a tool that can differentiate patients from controls according to type I interferon status, frequently in the absence of any other indices of inflammation.

**Fig. 4:**
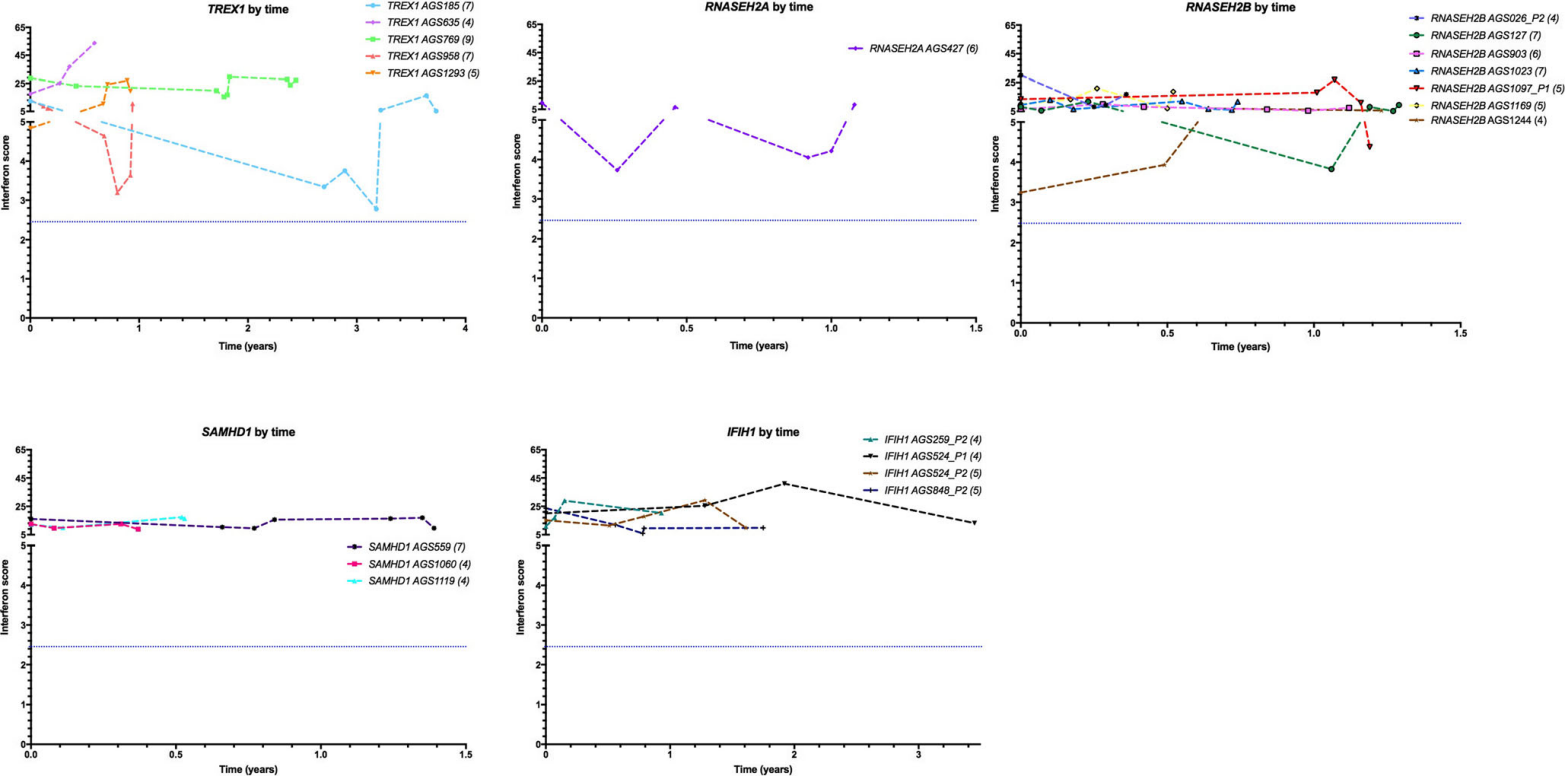
Interferon scores in patients where four or more serial samples were recorded. Data shown are interferon scores plotted against time (years) since first sampling. Interferon scores are calculated from the median fold change in relative quantification (RQ) values for a panel of six interferon-stimulated genes (ISGs). The *blue dashed line* represents the boundary of a positive/negative score (2.466). The number of serial samples for each patient is shown in *brackets* in the legend

Although ISG transcripts can be induced by infection, effectively resulting in a “false positive” result in the situations under consideration here, our data show that the IS is reproducible and consistent over time in the large majority of cases. Thus, taking all individuals in whom we recorded more than one IS, repeat sampling in 91 of 108 patients with a monogenic interferonopathy was consistent for a positive/negative IS (with nine of the 17 patients demonstrating discordant results being mutated in *RNASEH2B*). Furthermore, in 19 patients mutated in any of *AGS1-7* where we recorded four or more serial measurements, the scores were consistently positive in all cases—over periods spanning between 4 months and more than 3 years (Fig. 4). Indeed, we have shown previously that such repeat testing can enable the identification of new disease genes [18] and the definition of novel genotype-phenotype associations [19].

Our group 1 comprises 13 genotypes in which a link to enhanced interferon signaling seems established—*TREX1* [20], *RNASEH2A*, *RNASEH2B*, *RNASEH2C* [21], *SAMHD1* [22], *ADAR1* [16], *IFIH1* [18], *ACP5* [23, 24], *TMEM173* [25], *C1Q* [26], *C2*, *ISG15* [27], and *SKIV2L* [28]. Although we group these genotypes for ease of analysis, it is interesting that a comparison of the median RQ value for each of the six individual ISGs revealed markedly higher fold induction of *IFI27* and *SIGLEC1* in patients mutated in *ACP5*, *TMEM173*, *C1Q*, and *ISG15* compared to all other group 1 genotypes and to patients in group 3 (Fig. 3). This observation suggests genotype-specific patterns of ISG induction which are worthy of further interrogation, using genome-wide expression arrays, in a larger number of patients. We point out here that our cohort does not represent a survey of all putative monogenic type I interferonopathies, since we have yet to assess any patients with mutations in *PSMB8*, *PSMB4*, *PSMA3* [29], *DDX58* [30], *POLA1* [31], or *USP18* [32] using our screening assay [33].

In group 1, we measured 455 interferon signatures from 265 mutation-positive patients, of which 412 samples (91%) were abnormal. Of the 43 data points falling within the normal range, 33 were from patients mutated in *RNASEH2B* (Fig. 2, Table S26). As such, a normal result does not rule out a diagnosis of these discrete monogenic interferonopathies. However, a positive IS is clearly a reliable disease biomarker and can serve as a useful diagnostic screening tool.

The rationale for our group 2 designation was to try to identify further monogenic diseases demonstrating a consistent association with upregulated type I interferon signaling, where there is currently no biological evidence for such a link. For inclusion in this group, we required that at least one patient from each of at least two different families with the same monogenic disease demonstrated an upregulation of type I interferon signaling on at least one occasion. This allowed us to suggest that there might be a positive correlation of interferon induction with mutations in *CECR1* [34, 35], *RNASET2* [36, 37], *PRKDC* [38], *TRNT1* [39], and *DNASE1L3* [40, 41]. However, the small number of patients from whom we received repeat samples means that these putative associations need to be evaluated in larger cohorts of patients.

Mutations in the genes included in our group 1 can be associated with a remarkably broad spectrum of discrete or combined neurological, rheumatological, and dermatological presentations. Informed by these data, we identified a group of 24 patients from 17 families demonstrating a consistent upregulation of type I interferon signaling (median IS 9.38, IQR 6.67–13.98), all of whom tested negative for known clinically relevant type I interferonopathy-associated genes. Considering the occurrence of affected siblings in four of these families, there is a high likelihood that certain of these patients have a currently undefined genetic basis to their disease. Important clinical indicators that should prompt consideration of this type I interferonopathy grouping include vasculitic skin lesions, intracranial calcification, spasticity, dystonia, glaucoma, recurrent fevers, interstitial lung disease, and lupus-like disease.

We did not collect enough samples from any monogenic entities to make a definitive statement on a null relationship to type I upregulation. However, we did test a group of 41 patients clinically defined as having autoinflammatory disease, the majority of whom (27 of 41) showed no evidence of enhanced type I interferon signaling at any time (median IS 1.25, IQR 0.59–4.06). These data lend support to the specificity of type I interferon-induced gene transcript measurement as a screening tool and lead us to suggest that autoinflammation can be both interferon (e.g., due to mutations in *IFIH1* and *TMEM173*) and non-interferon related. Indeed, the only child included in our autoinflammatory group with a convincing upregulation of interferon signaling on multiple occasions (Table S23, AGS818) demonstrated recurrent chilblain-like lesions highly evocative of other type I interferonopathies.

While most patients with AGS are not currently treated by immunosuppression, a limitation of our study is that a majority of patients in groups 2 and 3 were receiving such therapy (details of which, where available, are given in S20, S21, S23, S24, and S25) when tested for an interferon signature. The possibility that such immunosuppression might attenuate a disease-associated upregulation of type I interferon signaling has been alluded to above. However, it is of note that many patients with JSLE and JDM demonstrated persistent upregulation of interferon signaling despite treatment. Interestingly, although sJIA is not normally associated with a type I interferon signature [42], we observed an upregulation of ISG expression in a small number of cases treated with IL1 blockers. This finding is concordant with a previous description of the induction of an interferon signature in JIA patients treated with anakinra and likely reflects currently undefined feedback loops triggered by these anti-cytokine agents [43]. As evidenced by the risk of developing interferon-driven pathology in the context of TNF-α blockade [44], such changes can be of clinical importance.

Summarizing, taken in clinical context, testing for an interferon signature represents a reliable screening tool for the identification of a variety of distinct genotypes and phenotypes. Such testing will likely become of high importance as therapies based on blocking interferon signaling become available [5, 45]. The interferon assay that we describe is practical, with the PAXgene system being stable for at least 72 h at room temperature, thus allowing for the easy transfer of samples to a reporting laboratory. At the same time, the IS represents a proxy assay, i.e., it does not directly measure the relevant disease-inducing molecule(s). Thus, we await the introduction of high-sensitivity assays of interferon protein which will be usefully combined with measures of ISG production as described here, thereby capturing the relationship between the inducing signal and the response to that signal.

## Electronic Supplementary Material

Below is the link to the electronic supplementary material.

ESM 1 (DOCX 1678 kb)
