# Supplementary material for: Assessment of Type I Interferon Signaling in Pediatric Inflammatory Disease
Source: J Clin Immunol. 2016 Dec 9;37(2):123–32. doi: 10.1007/s10875-016-0359-1 (PMC5325846; doi:10.1007/s10875-016-0359-1)
Supplement: Supplementary file 1 — (DOCX 1678 kb) [file 10875_2016_359_MOESM1_ESM.docx]

**Assessment of type I interferon signaling in pediatric inflammatory disease**

**Authors**

Gillian I Rice PhD^1^

Isabelle Melki MD^2,3,4,5^

Marie-Louise Frémond MD^2,3,5^

Tracy A Briggs PhD^1,6^

Mathieu P Rodero PhD^2,3^

Naoki Kitabayashi BSc^2,3^

Anthony Oojageer BSc^1^

Brigitte Bader-Meunier, MD^3,5,7^

Alexandre Belot, MD^8,9^

Christine Bodemer MD^3,10^

Pierre Quartier MD^3,5^

Yanick J Crow PhD^1,2,3^

**Table S1** Age, sex, and interferon score for 65 control individuals (78 measurements)

| **Control number** | **Age at sampling (Years)** | **Sex** | **Interferon score** |
| --- | --- | --- | --- |
| C1 | 12 | F | 1.605 |
| C2 | 38 | M | 0.331 |
| C3 | 22 | F | 0.379 |
| C4 | 23 | F | 0.691 |
| C5 | 24 | F | 0.684 |
| C6 | 27 | F | 2.162 |
| C7 | 34 | F | 0.772 |
| C8 | 35 | F | 1.289 |
| C9 | 35 | F | 1.309 |
| C10 | 37 | F | 1.230 |
| C10 | 39 | F | 0.538 |
| C11 | 43 | F | 2.561 |
| C12 | 16 | M | 0.446 |
| C13 | 6 | F | 0.590 |
| C14 | 43 | F | 0.953 |
| C14 | 43 | F | 0.558 |
| C15 | 58 | M | 1.068 |
| C15 | 59 | M | 1.284 |
| C16 | 1 | M | 1.185 |
| C17 | 10 | M | 1.486 |
| C18 | 13 | M | 0.663 |
| C19 | 2 | M | 0.931 |
| C20 | 22 | M | 0.507 |
| C21 | 30 | M | 0.478 |
| C22 | 37 | M | 0.349 |
| C23 | 4 | M | 0.774 |
| C24 | 13 | F | 0.545 |
| C25 | 40 | F | 1 |
| C26 | 37 | M | 3.167 |
| C27 | 48 | F | 0.768 |
| C28 | 12 | F | 2.221 |
| C29 | 4 | M | 0.925 |
| C30 | 44 | F | 6.793 |
| C31 | 42 | M | 0.558 |
| C31 | 42 | M | 0.972 |
| C32 | 76 | F | 0.894 |
| C33 | 79 | M | 3.054 |
| C34 | 42 | F | 0.939 |
| C34 | 42 | F | 1.112 |
| C34 | 42 | F | 1.079 |
| C35 | 26 | M | 0.370 |
| C36 | 30 | F | 1.615 |
| C37 | 34 | M | 2.676 |
| C38 | 63 | F | 0.793 |
| C39 | 30 | F | 0.387 |
| C40 | 93 | M | 1.135 |
| C41 | 29 | F | 1.468 |
| C42 | 47 | M | 0.417 |
| C42 | 47 | M | 1.916 |
| C43 | 42 | F | 0.486 |
| C43 | 42 | F | 0.692 |
| C44 | 1 | F | 0.847 |
| C45 | 35 | F | 0.281 |
| C46 | 42 | M | 0.576 |
| C47 | 38 | M | 0.341 |
| C48 | 38 | F | 1.851 |
| C49 | 43 | F | 0.285 |
| C50 | 50 | M | 1.724 |
| C51 | 50 | F | 0.204 |
| C51 | 50 | F | 0.388 |
| C52 | 35 | M | 0.467 |
| C53 | 35 | M | 0.623 |
| C54 | 83 | M | 0.595 |
| C54 | 83 | M | 0.476 |
| C54 | 83 | M | 0.612 |
| C55 | 67 | M | 0.276 |
| C55 | 67 | M | 0.187 |
| C55 | 67 | M | 0.353 |
| C56 | 31 | F | 0.497 |
| C57 | 34 | M | 0.535 |
| C58 | 65 | M | 2.567 |
| C59 | 39 | M | 23.4 |
| C59 | 39 | M | 0.34 |
| C60 | 39 | F | 0.826 |
| C61 | 37 | F | 0.872 |
| C62 | 27 | F | 0.299 |
| C63 | 55 | F | 0.345 |
| C64 | 28 | F | 0.411 |
| C65 | 38 | M | 0.397 |
| C65 | 38 | M | 0.280 |

M male; F female. C25 was chosen as the calibrator for the calculation of relative fold change, demonstrating RQ (relative quantification) values in the middle range for all 6 interferon stimulated genes. Values in pink are above the 2.466 cut-off.

**Table S2** Age, sex, interferon score and genotype for 76 parents and siblings (44 families, 89 measurements) who are heterozygous carriers of an AGS-related mutation

| **Patient number** | **Age at sampling (Years)** | **Sex** | **Interferon score** | **Mutation(s)** |
| --- | --- | --- | --- | --- |
| AGS026_M | 48.36 | M | 1.531 | *RNASEH2B* ND |
| AGS026_M | 48.72 | M | 1.672 | *RNASEH2B* ND |
| AGS026_F | 46.03 | F | 1.592 | *RNASEH2B* ND |
| AGS026_F | 46.39 | F | 2.842 | *RNASEH2B* ND |
| AGS044_M | 48.95 | M | 1.480 | *TREX1* ND |
| AGS044_F | 45.00 | F | 0.851 | *TREX1* ND |
| AGS067_F | 38.63 | F | 0.495 | *RNASEH2B* ND |
| AGS067_M | 39.33 | M | 0.843 | *RNASEH2B* ND |
| AGS068_M | 39.95 | M | 0.333 | *RNASEH2B* c.510+1 G>A het |
| AGS068_M | 40.83 | M | 0.475 | *RNASEH2B* c.510+1 G>A het |
| AGS068_F | 35.96 | F | 0.405 | *RNASEH2B* c.529G>A p.Ala177Thr het |
| AGS068_F | 36.84 | F | 0.556 | *RNASEH2B* c.529G>A p.Ala177Thr het |
| AGS070_F |  | F | 0.620 | *RNASEH2A* c.75C>T r.74_127del (p.Arg25Arg) het |
| AGS070_M |  | M | 0.336 | *RNASEH2A* c.704G>A p.Arg235Gln het |
| AGS081_M | 38.54 | M | 4.606 | *ADAR1* c.2675G>A p.Arg892His het |
| AGS081_M | 39.19 | M | 3.690 | *ADAR1* c.2675G>A p.Arg892His het |
| AGS081_F | 38.13 | F | 0.699 | *ADAR1* c.577C>G p.Pro193Ala het |
| AGS081_F | 38.78 | F | 1.101 | *ADAR1* c.577C>G p.Pro193Ala het |
| AGS093_F |  | F | 3.678 | *ADAR1* c.2608G>A p.Ala870Thr het |
| AGS093_M |  | M | 1.859 | *ADAR1* c.577C>G p.Pro193Ala het |
| AGS098_M |  | M | 0.449 | *RNASEH2B* c.529G>A p.Ala177Thr het |
| AGS098_F |  | F | 1.330 | *RNASEH2B* c.3C>A p.Met1Ile het |
| AGS107_F | 45.69 | F | 7.189 | *ADAR1* c.3337G>C p.Asp1113His het |
| AGS107_M | 46.77 | M | 0.431 | *ADAR1* c.3337G>C p.Asp1113His het |
| AGS128_F |  | F | 1.289 | *SAMHD1* c.445C>T p.Gln149* het |
| AGS160_F |  | F | 0.325 | *TREX1* c.341G>A p.Arg114His het |
| AGS171_F | 38.56 | F | 8.174 | *RNASEH2A* c.872G>A p.Arg291His het |
| AGS202_F | 49.13 | F | 0.875 | *RNASEH2A* c.635A>T p.Asn212Ile het |
| AGS202_M | 46.74 | M | 0.472 | *RNASEH2A* c.69G>A r.67_127del (p.Val23Val) het |
| AGS241_F |  | F | 0.409 | *ACP5* c.721G>A p.Asp241Asn het |
| AGS251_M |  | M | 0.482 | *ADAR1* ND |
| AGS251_F | 51 | F | 1.351 | *ADAR1* c.577C>G p.Pro193Ala het |
| AGS251_F | 52 | F | 2.018 | *ADAR1* c.577C>G p.Pro193Ala het |
| AGS262_F |  | F | 0.278 | *Acp5* c.667C>T p.Gln223* het |
| AGS266_M | 29.90 | M | 2.244 | *RNASEH2B* c.529G>A p.Ala177Thr het |
| AGS266_F | 31.12 | F | 0.892 | *RNASEH2B* c.529G>A p.Ala177Thr het |
| AGS301_F | 33.19 | F | 0.409 | *RNASEH2B* c.529G>A p.Ala177Thr het |
| AGS301_M | 48.53 | M | 1.236 | *RNASEH2B* c.529G>A p.Ala177Thr het |
| AGS305_F |  | F | 1.962 | *SAMHD1* c.1-6085_c.208+2691del p.Ex1del het |
| AGS327_M |  | M | 5.490 | *ADAR1* c.1076_1080del p.Lys359Argfs*14 het |
| AGS327_F |  | F | 1.158 | *ADAR1* c.577C>G p.Pro193Ala het |
| AGS368_M | 33 | M | 0.453 | *RNASEH2C* c.38G>A p.Arg13His het |
| AGS368_F | 32 | F | 0.673 | *RNASEH2C* c.38G>A p.Arg13His het |
| AGS427_M | 10.36 | M | 0.372 | *RNASEH2A* c.674A>G p.Glu225Gly het |
| AGS430_M | 33.01 | M | 0.720 | *ADAR1* c.2675G>A p.Arg892His het |
| AGS430_M | 33.79 | M | 12.498 | *ADAR1* c.2675G>A p.Arg892His het |
| AGS430_F | 33.52 | F | 23.022 | *ADAR1* c.577C>G p.Pro193Ala het |
| AGS430_F | 34.31 | F | 1.121 | *ADAR1* c.577C>G p.Pro193Ala het |
| AGS461_F | 36.27 | F | 1.921 | *Acp5* c.643G>A p.Gly215Arg het |
| AGS481_F | 44.95 | F | 1.162 | *Acp5* c.155A>C p.Lys52Thr het |
| AGS481_F | 26.61 | F | 1.130 | *Acp5* c.155A>C p.Lys52Thr het |
| AGS534_M | 33.60 | M | 0.584 | *RNASEH2B* c.263C>G p.Ala88Gly het |
| AGS534_M | 34.41 | M | 0.542 | *RNASEH2B* c.263C>G p.Ala88Gly het |
| AGS534_F | 29.75 | F | 0.712 | *RNASEH2B* c.31dup p.Val11Glyfs*32 het |
| AGS534_F | 30.57 | F | 3.110 | *RNASEH2B* c.31dup p.Val11Glyfs*32 het |
| AGS539_F | 23.79 | F | 0.562 | *RNASEH2B* c.529G>A p.Ala177Thr het |
| AGS540_F | 46.59 | F | 1.005 | *RNASEH2B* c.529G>A p.Ala177Thr het |
| AGS540_M | 47.94 | M | 0.286 | *RNASEH2B* c.529G>A p.Ala177Thr het |
| AGS540_F |  | F | 62.563 | *RNASEH2B* c.529G>A p.Ala177Thr het |
| AGS581_F | 44.89 | F | 0.586 | *RNASEH2A* c.323G>A r.Arg108del het |
| AGS584_M | 39.49 | M | 0.838 | *SAMHD1* c.703C>T p.Gln235* het |
| AGS584_F | 37.86 | F | 1.017 | *SAMHD1* c.703C>T p.Gln235* het |
| AGS628_M | 22.73 | M | 18.288 | *SAMHD1* c.1063G>A p.Glu355Lys het; 1x*SAMHD1* ex5, 3x*SAMHD1* ex6 by MLPA |
| AGS628_F | 21.93 | F | 0.862 | *SAMHD1* 1xSAMHD1 ex1, ex2 by MLPA |
| AGS631_F | 37 | F | 0.766 | *Acp5* c.155A>C p.Lys52Thr het; c.790A>G p.Met264Val het |
| AGS631_M | 37.30 | M | 0.418 | *Acp5* c.155A>C p.Lys52Thr het |
| AGS688_M | 21.77 | M | 0.576 | *RNASEH2B* c.529G>A p.Ala177Thr het |
| AGS720_F | 37.24 | F | 0.686 | *ADAR1* c.577C>G p.Pro193Ala het |
| AGS785_M | 66.55 | M | 0.659 | *CECR1* c.578C>T p.Pro193Leu het |
| AGS785_F | 64.34 | F | 0.474 | *CECR1* c.506G>A p.Arg169Gln het |
| AGS793_M |  | M | 0.491 | *RNASEH2B* c.529G>A p.Ala177Thr het |
| AGS793_F |  | F | 0.511 | *RNASEH2B* c.529G>A p.Ala177Thr het |
| AGS848_F | 41.59 | F | 0.407 | *RNASEH2B* c.529G>A p.Ala177Thr het |
| AGS848_F | 41.59 | F | 0.274 | *RNASEH2B* c.529G>A p.Ala177Thr het |
| AGS850_F | 37.19 | F | 5.982 | *SAMHD1* c.1411-2A>G het |
| AGS850_M | 43.83 | M | 0.744 | *SAMHD1* c.1411-2A>G het |
| AGS850_F | 39.01 | F | 0.999 | *SAMHD1* c.1411-2A>G het |
| AGS978_F | 42.02 | F | 2.927 | *SAMHD1* c.509G>A p.Gly170Glu het |
| AGS978_M | 44.57 | M | 0.523 | *SAMHD1* c.509G>A p.Gly170Glu het |
| AGS1060_F | 40.65 | F | 8.133 | *SAMHD1* c.386A>G p.His129Arg het |
| AGS1060_F | 40.88 | F | 4.313 | *SAMHD1* c.386A>G p.His129Arg het |
| AGS1060_M | 42.23 | M | 0.171 | *SAMHD1* c.602T>A p.Ile201Asn het |
| AGS1119_M | 0.74 | M | 0.495 | *SAMHD1* c.428G>A p.Arg143His het |
| AGS1119_M | 2.01 | M | 0.872 | *SAMHD1* c.428G>A p.Arg143His het |
| AGS1170_F | 18.70 | F | 0.444 | *ADAR1* ND |
| AGS1170_M |  | M | 11.794 | *ADAR1* ND |
| AGS1315_M | 30.18 | M | 2.692 | *ADAR1* c.3019G>A p.Gly1007Arg mosaic |
| AGS1507_M | 36.77 | M | 1.672 | *ADAR1* c.2763-2A>G het |
| AGS1507_F | 38.28 | F | 3.169 | *ADAR1* c.577C>G p.Pro193Ala het |

M male; F female; het heterozygous; hom homozygous. ND Not determined (parents of children with biallelic mutations, where parental genotype has not been formally tested). Values in pink are above the 2.466 cut-off.

**Table S3** Age, sex, interferon score and genotype for 40 patients (35 families, 77 measurements) with mutations in *TREX1*

| **Patient number** | **Age at sampling (Years)** | **Sex** | **Phenotype** | **Interferon score** | **Mutation(s)** |
| --- | --- | --- | --- | --- | --- |
| AGS044 | 13.21 | M | AGS | 30.999 | c.341 G>A p.Arg114His het; c.598_600dup p.Asp200dup het |
| AGS058 | 15.73 | M | AGS | 5.207 | c.397del p.Leu133Cysfs*27 hom |
| AGS060 | 12.93 | M | AGS | 12.954 | c.598 G>A p.Asp200Asn het *de novo* |
| AGS160 | 6.7 | F | AGS | 4.709 | c.341 G>A p.Arg114His hom |
| AGS161 | 11.13 | F | AGS | 14.455 | c.868_885del p.Pro290_Ala295del hom |
| AGS161 | 12.47 | F |  | 8.123 |  |
| AGS185 | 6.48 | M | AGS | 12.393 | c.868_885del p.Pro290_Ala295del hom |
| AGS185 | 9.18 | M |  | 3.343 |  |
| AGS185 | 9.37 | M |  | 3.754 |  |
| AGS185 | 9.66 | M |  | 2.775 |  |
| AGS185 | 9.70 | M |  | 5.929 |  |
| AGS185 | 10.12 | M |  | 16.341 |  |
| AGS185 | 10.21 | M |  | 5.260 |  |
| AGS195 | 12.17 | F | AGS | 6.652 | c.341G>A p.Arg114His het; c.485T>C p.Leu162Pro het |
| AGS195 | 12.17 | F |  | 6.483 |  |
| AGS195 | 12.55 | F |  | 11.277 |  |
| AGS205 | 22.07 | F | AGS | 20.360 | c.52 G>A p.Asp18Asn het *de novo* |
| AGS205 | 23.38 | F |  | 15.456 |  |
| AGS300 | 7.71 | M | AGS | 20.953 | c.341 G>A p.Arg114His hom |
| AGS350 | 6.34 | F | AGS | 18.187 | c.393_408dup p.Glu137fs hom |
| AGS441 | 4.64 | F | AGS | 19.791 | c.868_885del p.Pro290_Ala295del hom |
| AGS525 | 3.5 | F | AGS | 1.542 | c.152_153del p.Gln51Argfs*50 het; c.197 A>G p.Lys66Arg het |
| AGS525 | 3.5 | F |  | 2.439 |  |
| AGS535 | 0.75 | M | AGS | 12.649 | c.635del p.Pro212Hisfs*65 hom |
| AGS535 | 1.08 | M |  | 28.759 |  |
| AGS565 | 0.72 | F | AGS | 35.481 | c.294dup p.Cys99Metfs*3 hom |
| AGS574 | 0.39 | M | AGS | 18.689 | c.58dup p.Glu20Glyfs*82 het; c.625_628dup p.Trp210Serfs*32 het |
| AGS635 | 1.74 | M | AGS | 17.558 | c.52 G>A p.Asp18Asn het *de novo* |
| AGS635 | 2.01 | M |  | 25.086 |  |
| AGS635 | 2.10 | M |  | 36.950 |  |
| AGS635 | 2.33 | M |  | 53.744 |  |
| AGS650_P1 | 12.22 | M | AGS | 7.728 | c.868_885del p.Pro289_Ala295del hom |
| AGS650_P2 | 8.22 | M | AGS | 3.294 | c.868_885del p.Pro289_Ala295del hom |
| AGS652 | 6.1 | M | AGS | 10.888 | c.868_885del p.Pro289_Ala295del hom |
| AGS653 | 4.05 | M | AGS | 10.505 | c.237_243del p.Ala81Argfs*5 hom |
| AGS769 | 12.91 | M | AGS | 28.715 | c.341G>A p.Arg114His het; c.634del p.Pro212Hisfs*65 het |
| AGS769 | 13.33 | M |  | 23.257 |  |
| AGS769 | 14.62 | M |  | 19.869 |  |
| AGS769 | 14.69 | M |  | 15.485 |  |
| AGS769 | 14.72 | M |  | 16.849 |  |
| AGS769 | 14.74 | M |  | 29.629 |  |
| AGS769 | 15.27 | M |  | 27.874 |  |
| AGS769 | 15.30 | M |  | 23.791 |  |
| AGS769 | 15.35 | M |  | 27.223 |  |
| AGS804_P1 | 1.77 | F | FCL | 19.783 | c.52G>A p. Asp18Asn het |
| AGS804_P1 | 2.20 | F |  | 14.287 |  |
| AGS804_P1 | 3.58 | F |  | 17.264 |  |
| AGS804_P2 | 34.34 | F | FCL | 8.784 | c.52G>A p. Asp18Asn het |
| AGS804_P2 | 35.72 | F |  | 5.828 |  |
| AGS955 | 0.59 | F | AGS | 16.717 | c.144dup C p.Thr49Hisfs*50 hom |
| AGS958 | 0.31 | F | AGS | 12.951 | c.341G>A p.Arg114His het; c.907A>C p.Thr303Pro het |
| AGS958 | 0.43 | F |  | 8.819 |  |
| AGS958 | 0.47 | F |  | 7.286 |  |
| AGS958 | 0.99 | F |  | 4.643 |  |
| AGS958 | 1.11 | F |  | 3.197 |  |
| AGS958 | 1.23 | F |  | 3.641 |  |
| AGS958 | 1.25 | F |  | 10.632 |  |
| AGS972 | 0.74 | F | AGS | 11.182 | c.182C>A p.Pro61Gln hom |
| AGS1003_P1 | 2.52 | M | FCL | 2.796 | c.52G>C p.Asp18His het |
| AGS1003_P2 | 31.13 | F | FCL + polyarthralgia | 5.099 | c.52G>C p.Asp18His het |
| AGS1021 | 1.36 | M | AGS | 10.729 | c.206T>C p.Leu69Pro hom |
| AGS1070 | 0.14 | M | AGS | 27.20 | c.45C>G p.Ile15Met hom |
| AGS1081_P1 | 27.68 | F | AGS | 6.194 | c.598G>A p.Asp200Asn het |
| AGS1081_P2 | 49.14 | F | FCL | 7.727 | c.598G>A p.Asp200Asn het |
| AGS1082 | 0.88 | M | AGS | 8.03 | c.341G>A p.Arg114His het + c.416delC p.Ala139Valfs*21 het |
| AGS1085_P1 | 14.65 | M | FCL | 33.831 | c.599A>C p.Asp200Ala het§ |
| AGS1085_P2 | 35.67 | F | Clinically unaffected | 3.161 | c.599A>C p.Asp200Ala het§ |
| AGS1116 | 6.14 | F | AGS | 3.946 | c.52G>A p.Asp18Asn het |
| AGS1116 | 6.19 | F |  | 5.889 |  |
| AGS1116 | 6.23 | F |  | 0.568 |  |
| AGS1266 | 21.17 | M | AGS | 8.075 | c.598G>A p.Asp200Asn het^ Inheritance not determined |
| AGS1284 | 0.04 | M | AGS | 8.633 | c.341G>A p.Arg114His hom |
| AGS1293 | 0.19 | M | AGS | 4.839 | c.341G>A p.Arg114His het; c.622_626del p.Cys208Valfs*31 het |
| AGS1293 | 0.86 | M |  | 10.399 |  |
| AGS1293 | 0.90 | M |  | 24.219 |  |
| AGS1293 | 1.08 | M |  | 26.951 |  |
| AGS1293 | 1.11 | M |  | 19.49 |  |
| AGS1662 | 1.26 | F | AGS | 8.763 | c.341G>A p.Arg114His hom |

M male; F female; het heterozygous; hom homozygous; AGS Aicardi-Goutières syndrome; FCL familial chilblain lupus. Nucleotide numbering based on transcript *TREX1* NM_033629.2. Protein numbering based on transcript NP_338599.1. §Mother and Son with *ADAR1* c.3463C>T p.Arg1155Trp het variant in addition to *TREX1* variant. AGS1116 variant inherited from Dad. ^Also has *IFIH1* c.2192C>T p.Ala731Val het. Values in green are below the 2.466 cut-off.

**Table S4** Age, sex, interferon score and genotype for 5 patients (5 families, 11 measurements) with mutations in *RNASEH2A*

| **Patient number** | **Age at sampling (Years)** | **Sex** | **Phenotype** | **Interferon score** | **Mutation(s)** |
| --- | --- | --- | --- | --- | --- |
| AGS070 | 14.01 | F | AGS | 19.591 | c.704 G>A p.Arg235Gln het; c.75 C>T p.Arg25Arg het |
| AGS171 | 5.89 | F | AGS | 17.356 | c.207dup p.Thr70Aspfs*50 het; c.872 G>A p.Arg291His het |
| AGS171 | 7.02 | F |  | 43.812 |  |
| AGS202 | 12.52 | M | AGS | 4.233 | c.69 G>A p.Val23Val het; c.635 A>T p.Asn212Ile het |
| AGS427 | 5.76 | M | AGS | 9.714 | c.674A>G p.Glu225Gly hom |
| AGS427 | 6.02 | M |  | 3.729 |  |
| AGS427 | 6.22 | M |  | 6.519 |  |
| AGS427 | 6.68 | M |  | 4.051 |  |
| AGS427 | 6.76 | M |  | 4.215 |  |
| AGS427 | 6.84 | M |  | 8.303 |  |
| AGS581 | 7.73 | M | AGS | 39.630 | c.206dup p.Thr70Aspfs*50 hom |

M male; F female; het heterozygous; hom homozygous; AGS Aicardi-Goutières syndrome. Nucleotide numbering based on transcript *RNASEH2A* NM_006397.2. Protein numbering based on transcript NP_006388.2.

**Table S5** Age, sex, interferon score and genotype for 84 patients (73 families, 148 measurements) with mutations in *RNASEH2B*

| **Patient number** | **Age at sampling (Years)** | **Sex** | **Phenotype** | **Interferon score** | **Mutation(s)** |
| --- | --- | --- | --- | --- | --- |
| AGS011_P1 | 8.15 | M | AGS | 6.921 | c.257A>G p.His86Arg het; c.529G>A p.Ala177Thr het |
| AGS011_P1 | 8.33 | M |  | 7.13 |  |
| AGS011_P1 | 8.44 | M |  | 5.253 |  |
| AGS011_P2 | 12.63 | M | AGS | 5.831 | c.257A>G p.His86Arg het; c.529G>A p.Ala177Thr het |
| AGS011_P2 | 12.81 | M |  | 7.846 |  |
| AGS011_P2 | 12.92 | M |  | 5.129 |  |
| AGS015 | 12.01 | M | AGS | 2.149 | c.510+1 G>A het; c.529 G>A p.Ala177Thr het |
| AGS023 | 15.31 | M | AGS | 12.497 | c.436+1 G>T het; c.529G>A p.Ala177Thr het |
| AGS026_P1 | 20.35 | M | AGS | 1.668 | c.136+1 del het; c.529 G>A p.Ala177Thr het |
| AGS026_P1 | 20.64 | M |  | 4.495 |  |
| AGS026_P1 | 20.71 | M |  | 4.401 |  |
| AGS026_P2 | 16.63 | M | AGS | 30.048 | c.136+1 del het; c.529 G>A p.Ala177Thr het |
| AGS026_P2 | 16.88 | M |  | 8.109 |  |
| AGS026_P2 | 16.92 | M |  | 9.457 |  |
| AGS026_P2 | 16.99 | M |  | 16.887 |  |
| AGS049 | 11.59 | M | AGS | 0.699 | c.529 G>A p.Ala177Thr hom |
| AGS067 | 8.24 | F | AGS | 1.050 | c.529 G>A p.Ala177Thr het; c.128 C>A p.Pro43His het |
| AGS068 | 7.73 | F | AGS | 10.371 | c.510+1 G>A het; c.529 G>A p.Ala177Thr het |
| AGS068 | 8.05 | F |  | 5.220 |  |
| AGS068 | 8.61 | F |  | 8.163 |  |
| AGS071_P1 | 15.36 | F | AGS | 13.102 | c.554T>G p.Val185Gly hom |
| AGS071_P2 | 0.45 | F | AGS | 11.485 | c.554T>G p.Val185Gly hom |
| AGS071_P2 | 0.47 | F |  | 7.579 |  |
| AGS098_P1 | 29.13 | F | AGS | 12.853 | c.529 G>A p.Ala177Thr het; c.3 G>A p.Met1? het |
| AGS098_P2 | 23.24 | F | AGS | 15.427 | c.529 G>A p.Ala177Thr het; c.3 G>A p.Met1? het |
| AGS119 | 11.39 | M | AGS | 5.418 | c.136+1delG het; c.529G>A p.Ala177Thr het |
| AGS119 | 11.46 | M |  | 6.989 |  |
| AGS127 | 8.42 | M | AGS | 8.13 | c.488C>T p.Thr163Ile het; c.529G>A p.Ala177Thr het |
| AGS127 | 8.49 | M |  | 5.44 |  |
| AGS127 | 8.65 | M |  | 11.762 |  |
| AGS127 | 9.48 | M |  | 3.827 |  |
| AGS127 | 9.61 | M |  | 8.029 |  |
| AGS127 | 9.69 | M |  | 5.211 |  |
| AGS127 | 9.71 | M |  | 9.379 |  |
| AGS134 | 17.94 | M | AGS | 6.011 | c.529G>A p.Ala177Thr hom |
| AGS140_P1 | 21.13 | M | AGS | 9.396 | c.244+1G>T het; c.529G>A p.Ala177Thr het |
| AGS140_P2 | 28.61 | M | AGS | 18.264 | c.244+1G>T het; c.529G>A p.Ala177Thr het |
| AGS144 | 13.26 | F | AGS | 1.48 | c.529G>A p.Ala177Thr hom |
| AGS144 | 13.34 | F |  | 0.63 |  |
| AGS144 | 13.49 | F |  | 0.927 |  |
| AGS146 | 15.96 | M | AGS | 35.133 | c.529G>A p.Ala177Thr hom |
| AGS146 | 16.11 | M |  | 11.395 |  |
| AGS190 | 10.02 | F | AGS | 5.423 | c.529G>A p.Ala177Thr het; c.412C>T p.Leu138Phe het |
| AGS199 | 0.64 | M | AGS | 5.566 | c.529G>A p.Ala177Thr het; Ex2-5del p.Ex2_5del het |
| AGS220 | 6.4 | F | AGS | 0.718 | c.136+1 del het; c.529 G>A p.Ala177Thr het |
| AGS230_P1 | 13.78 | M | AGS | 0.656 | c.529G>A p.Ala177Thr hom |
| AGS230_P2 | 15.67 | M | AGS | 0.568 | c.529G>A p.Ala177Thr hom |
| AGS231 | 9.25 | F | AGS | 2.355 | c.529G>A p.Ala177Thr het; c.634G>A p.Ala212Thr het |
| AGS233 | 8.36 | M | SP | 8.903 | c.529G>A p.Ala177Thr hom |
| AGS238 | 6.12 | F | AGS | 3.616 | c.529G>A p.Ala177Thr hom |
| AGS252 | 5.29 | M | AGS | 4.223 | c.529G>A p.Ala177Thr hom |
| AGS252 | 7.31 | M |  | 2.735 |  |
| AGS266 | 5.19 | F | AGS | 61.764 | c.529G>A p.Ala177Thr hom |
| AGS297 | 7.99 | M | AGS | 2.374 | c.529G>A p.Ala177Thr hom |
| AGS301 | 6.26 | M | AGS | 2.329 | c.529G>A p.Ala177Thr hom |
| AGS301 | 6.37 | M |  | 1.518 |  |
| AGS304 | 10.37 | F | AGS | 1.558 | c.488C>T p.Thr163Ile het; c.529G>A p.Ala177Thr het |
| AGS442_P1 | 5.77 | F | AGS | 4.242 | c.529G>A p.Ala177Thr het; c.616 +1 G>A het |
| AGS442_P2 | 4.31 | F | AGS | 8.892 | c.529G>A p.Ala177Thr het; c.616 +1 G>A het |
| AGS486 | 2.36 | F | AGS | 13.198 | c.529G>A p.Ala177Thr hom |
| AGS486 | 4.03 | F |  | 2.437 |  |
| AGS533 | 19.07 | M | AGS | 11.142 | c.529G>A p.Ala177Thr hom |
| AGS534 | 0.96 | F | AGS | 11.020 | c.263C>G p.Ala88Gly het; c.31dup p.Val11Glyfs*32 het |
| AGS539 | 3.35 | M | AGS | 1.635 | c.529G>A p.Ala177Thr hom |
| AGS539 | 3.66 | M |  | 2.638 |  |
| AGS540 | 15.04 | F | AGS | 4.931 | c.529G>A p.Ala177Thr hom |
| AGS540 | 15.34 | F |  | 6.318 |  |
| AGS540 | 15.37 | F |  | 5.196 |  |
| AGS558 | 3.14 | F | AGS | 1.299 | c.529G>A p.Ala177Thr hom |
| AGS560 | 1.1 | F | AGS | 7.363 | c.529G>A p.Ala177Thr hom |
| AGS580_P1 | 8.14 | M | AGS | 8.584 | c.179T>G p.Leu60Arg het; c.529G>A p.Ala177Thr het |
| AGS580_P1 | 8.18 | M |  | 12.372 |  |
| AGS580_P2 | 2.08 | M | AGS | 14.025 | c.179T>G p.Leu60Arg het; c.529G>A p.Ala177Thr het |
| AGS627 | 2.05 | F | AGS | 7.001 | c.529G>A p.Ala177Thr het; c.488C>T p.Thr163Ile het |
| AGS627 | 2.59 | F |  | 7.731 |  |
| AGS648 | 3.21 | M | AGS | 6.107 | c.529G>A p.Ala177Thr hom |
| AGS651 | 2.89 | M | AGS | 34.129 | c.554T>G p.Val185Gly hom |
| AGS673 | 4.63 | M | AGS | 2.769 | c.529G>A p.Ala177Thr hom |
| AGS673 | 4.99 | M |  | 1.709 |  |
| AGS688 | 1.63 | M | AGS | 5.424 | c.529G>A p.Ala177Thr hom |
| AGS688 | 2.02 | M |  | 2.398 |  |
| AGS689 | 0.36 | M | AGS | 12.003 | c.554T>G p.Val185Gly hom |
| AGS689 | 1.30 | M |  | 4.296 |  |
| AGS689 | 2.61 | M |  | 12.581 |  |
| AGS690 | 0.59 | M | AGS | 5.124 | c.529G>A p.Ala177Thr hom |
| AGS707 | 8.81 | M | AGS | 25.741 | c.529G>A p.Ala177Thr hom |
| AGS707 | 8.94 | M |  | 1.583 |  |
| AGS711 | 2.82 | F | SP | 4.031 | c.529G>A p.Ala177Thr hom |
| AGS711 | 3.31 | F |  | 1.312 |  |
| AGS739_P1 | 7.16 | F | SP | 2.987 | c.529G>A p.Ala177Thr hom |
| AGS739_P2 | 10.66 | F | SP | 1.337 | c.529G>A p.Ala177Thr hom |
| AGS760 | 6.69 | F | AGS | 2.065 | c.488C>T p.Thr163Ile; c.844A>T p.Lys295Ile |
| AGS768 | 5.69 | M | SP | 1.900 | c.529G>A p.Ala177Thr hom |
| AGS793 | 11.26 | M | AGS | 0.242 | c.529G>A p.Ala177Thr hom |
| AGS825 | 4.84 | F | AGS | 0.640 | c.529G>A p.Ala177Thr hom |
| AGS825 | 4.91 | F |  | 0.960 |  |
| AGS834 | 6.39 | F | AGS | 0.709 | c.529G>A p.Ala177Thr het; c.412C>T p.Leu138Phe het |
| AGS869 | 3.89 | F | AGS | 11.035 | c.529G>A p.Ala177Thr hom |
| AGS903 | 2.95 | F | AGS | 6.584 | c.226A>T p.Asn76Tyr het; c.529G>A p.Ala177Thr het |
| AGS903 | 3.23 | F |  | 9.953 |  |
| AGS903 | 3.37 | F |  | 8.113 |  |
| AGS903 | 3.79 | F |  | 6.311 |  |
| AGS903 | 3.93 | F |  | 5.387 |  |
| AGS903 | 4.07 | F |  | 7.447 |  |
| AGS912 | 2.82 | F | AGS | 8.908 | c.229C>T p.Gln77* het; c.529G>A p.Ala177Thr het |
| AGS973 | 9.78 | F | AGS | 13.765 | c.529G>A p.Ala177Thr hom |
| AGS981 | 6.42 | M | AGS | 11.323 | c.529G>A p.Ala177Thr hom |
| AGS1023 | 0.39 | M | AGS | 9.408 | c.529G>A p.Ala177Thr hom§ |
| AGS1023 | 0.49 | M |  | 12.919 |  |
| AGS1023 | 0.57 | M |  | 6.512 |  |
| AGS1023 | 0.94 | M |  | 12.02 |  |
| AGS1023 | 1.03 | M |  | 6.737 |  |
| AGS1023 | 1.11 | M |  | 6.022 |  |
| AGS1023 | 1.13 | M |  | 11.716 |  |
| AGS1046 | 1.35 | F | AGS | 10.513 | c.529G>A p.Ala177Thr hom |
| AGS1046 | 2.09 | F |  | 2.531 |  |
| AGS1061 | 1.59 | F | AGS | 3.539 | c.529G>A p.Ala177Thr hom |
| AGS1061 | 1.93 | F |  | 4.355 |  |
| AGS1069 | 2.62 | F | AGS | 16.331 | c.529G>A p.Ala177Thr hom |
| AGS1097_P1 | 2.14 | F | AGS | 13.481 | c.529G>A p.Ala177Thr hom |
| AGS1097_P1 | 3.15 | F |  | 18.011 |  |
| AGS1097_P1 | 3.21 | F |  | 26.838 |  |
| AGS1097_P1 | 3.30 | F |  | 10.946 |  |
| AGS1097_P1 | 3.33 | F |  | 4.379 |  |
| AGS1097_P2 | 4.88 | M | AGS | 0.451 | c.529G>A p.Ala177Thr hom |
| AGS1097_P2 | 5.95 | M |  | 54.887 |  |
| AGS1122 | 15.45 | M | AGS | 2.418 | c.529G>A p.Ala177Thr het; c.511-2A>G het |
| AGS1169 | 1.46 | M | AGS | 10.063 | c.529G>A p.Ala177Thr het; c.685T>C p.Ser229Pro het |
| AGS1169 | 1.63 | M |  | 13.327 |  |
| AGS1169 | 1.72 | M |  | 20.92 |  |
| AGS1169 | 1.96 | M |  | 7.017 |  |
| AGS1169 | 1.98 | M |  | 18.702 |  |
| AGS1244 | 0.34 | M | AGS | 3.241 | c.529G>A p.Ala177Thr hom |
| AGS1244 | 0.83 | M |  | 3.935 |  |
| AGS1244 | 0.98 | M |  | 7.03 |  |
| AGS1244 | 1.57 | M |  | 5.565 |  |
| AGS1332 | 1.03 | M | AGS | 4.455 | c.529G>A p.Ala177Thr hom |
| AGS1438 | 0.83 | F | AGS | 9.28 | c.529G>A p.Ala177Thr hom |
| AGS1502 | 8.53 | M | AGS | 3.236 | c.529G>A p.Ala177Thr hom |
| AGS1518 | 1.18 | M | AGS | 25.299 | c.529G>A p.Ala177Thr hom |
| AGS1518 | 1.31 | M |  | 5.647 |  |
| AGS1522_P1 | 12.57 | F | AGS | 1.644 | c.529G>A p.Ala177Thr hom |
| AGS1522_P2 | 17.61 | F | AGS | 2.534 | c.529G>A p.Ala177Thr hom |
| AGS1525 | 4.60 | M | AGS | 2.018 | c.529G>A p.Ala177Thr hom |
| AGS1535 | 1.43 | F | AGS | 6.094 | c.529G>A p.Ala177Thr hom |
| AGS1535 | 1.73 | F |  | 10.173 |  |
| AGS1586 | 3.26 | F | AGS | 3.288 | c.529G>A p.Ala177Thr hom |
| AGS1785 | 7.85 | M | AGS | 6.477 | c.529G>A p.Ala177Thr hom |

M male; F female; het heterozygous; hom homozygous; AGS Aicardi-Goutières syndrome; SP spastic paraparesis. Nucleotide numbering based on transcript *RNASEH2B* NM_024570.3. Protein numbering based on transcript NP_078846.2. §*RNASEH2B* c.529G>A p.Ala177Thr hom + *IFIH1* c.1075G>C p.Val359Leu het. Values in green are below the 2.466 cut-off.

**Table S6** Age, sex, interferon score and genotype for 13 patients (11 families, 16 measurements) with mutations in *RNASEH2C*

| **Patient number** | **Age at sampling (Years)** | **Sex** | **Phenotype** | **Interferon score** | **Mutation(s)** |
| --- | --- | --- | --- | --- | --- |
| AGS037 | 9.8 | M | AGS | 15.523 | c.205 C>T p.Arg69Trp hom |
| AGS088_P1 | 10.95 | M | AGS | 8.631 | c.205 C>T p.Arg69Trp hom |
| AGS088_P1 | 11.46 | M |  | 14.117 |  |
| AGS088_P2 | 16.15 | M | AGS | 9.765 | c.205 C>T p.Arg69Trp hom |
| AGS088_P2 | 16.65 | M |  | 16.297 |  |
| AGS234 | 7.15 | F | AGS | 6.017 | c.115 G>T p.Asp39Tyr het; c.173-1G>C het |
| AGS368 | 8.81 | F | AGS | 6.838 | c.38 G>A p.Arg13His hom |
| AGS548_P1 | 3.87 | F | Clinically unaffected | 6.813 | c.205 C>T p.Arg69Trp hom |
| AGS548_P2 | 5.7 | F | AGS | 4.053 | c.205 C>T p.Arg69Trp hom |
| AGS696 | 1.77 | F | AGS | 9.714 | c.38G>A p.Arg13His hom |
| AGS733 | 1.95 | M | AGS | 7.720 | c.215_218delinsTGGC p.Glu72_Val73delins ValAla hom |
| AGS771 | 0.07 | M | AGS | 8.739 | c.205 C>T p.Arg69Trp hom |
| AGS827 | 0.01 | F | AGS | 9.469 | c.205 C>T p.Arg69Trp hom |
| AGS1100 | 7.76 | M | AGS | 14.472 | c.485C>T p.Pro162Leu hom |
| AGS1503 | 0.59 | F | AGS | 12.773 | c.205 C>T p.Arg69Trp hom |
| AGS1503 | 0.95 | F |  | 5.716 |  |

M male; F female; het heterozygous; hom homozygous; AGS Aicardi-Goutières syndrome. Nucleotide numbering based on transcript *RNASEH2C* NM_032193.3. Protein numbering based on transcript NP_115569.2.

**Table S7** Age, sex, interferon score and genotype for 31 patients (25 families, 45 measurements) with mutations in *SAMHD1*

| **Patient number** | **Age at sampling (Years)** | **Sex** | **Phenotype** | **Interferon score** | **Mutation(s)** |
| --- | --- | --- | --- | --- | --- |
| AGS079 | 16.39 | F | AGS | 30.730 | c.433 C>T p.Arg145* hom |
| AGS084 | 25.98 | M | AGS | 5.951 | c.1609-1G>C hom^ |
| AGS104_P1 | 13.25 | M | AGS | 46.955 | c.433 C>T p.Arg145* hom |
| AGS104_P2 | 12.16 | F | AGS | 14.289 | c.433 C>T p.Arg145* hom |
| AGS116 | 32.84 | M | AGS | 10.593 | c.649_650insG p.Phe217Cysfs*2 het; c.1-6085_c.208+2691del het |
| AGS126 | 7.94 | F | AGS | 6.152 | c.428 G>A p.Arg143His hom |
| AGS128 | 6.77 | F | AGS | 14.200 | c.445 C>T p.Gln149* hom |
| AGS128 | 7.02 | F |  | 15.899 |  |
| AGS128 | 7.06 | F |  | 9.761 |  |
| AGS145 | 19.97 | M | SP | 21.333 | c.1642 C>T p.Gln548* hom |
| AGS165 | 4.54 | F | AGS | 12.533 | c.359_370 del p.Asp120_His123del hom |
| AGS282_P1 | 15.48 | F | Inflammatory | 10.682 | c.433 C>T p.Arg145* het; c.490 C>T p.Arg164* het |
| AGS282_P2 | 8.48 | M | Spasticity | 12.151 | c.433 C>T p.Arg145* het; c.490 C>T p.Arg164* het |
| AGS305 | 9.33 | F | AGS | 13.632 | c.1-6085_c.208+2691del hom |
| AGS348 | 15.14 | M | AGS | 60.985 | c.1476del p.Lys492Asnfs*2 het; c.1-6085_c.208+2691del het |
| AGS449 | 11.69 | M | AGS | 14.282 | c.433 C>T p.Arg145* hom |
| AGS495 | 1.27 | F | AGS | 10.004 | c.1609-1G>C hom |
| AGS559 | 10.69 | M | AGS | 16.156 | c.868C>T p.Arg290Cys hom |
| AGS559 | 11.35 | M |  | 10.34 |  |
| AGS559 | 11.46 | M |  | 9.569 |  |
| AGS559 | 11.53 | M |  | 15.593 |  |
| AGS559 | 11.93 | M |  | 16.361 |  |
| AGS559 | 12.04 | M |  | 16.904 |  |
| AGS584 | 5.33 | F | AGS | 8.278 | c.703C>T p.Gln235* hom |
| AGS649 | 8.74 | F | AGS | 9.621 | c.1324 C>T p.Arg442* hom |
| AGS682 | 0.35 | F | AGS | 6.094 | c.1-6085_c.208+2691del p.Ex1del hom |
| AGS757 | 2.96 | M | AGS | 7.866 | c.1693G>A p.Ala565Thr hom§ |
| AGS850_P1 | 5.50 | F | AGS | 5.637 | c.1411-2A>G hom |
| AGS850_P2 | 15.78 | F | FCL | 16.66 | c.1411-2A>G hom |
| AGS850_P3 | 2.18 | M | AGS | 16.911 | c.1411-2A>G hom |
| AGS850_P4 | 20.82 | M | AGS | 24.483 | c.1411-2A>G hom |
| AGS978_P1 | 4.05 | M | AGS | 17.918 | c.509G>A p.Gly170Glu hom |
| AGS978_P2 | 18.00 | F | AGS | 35.077 | c.509G>A p.Gly170Glu hom |
| AGS1060 | 10.94 | M | FCL | 12.506 | c.386A>G p.His129Arg het; c.602T>A p.Ile201Asn het |
| AGS1060 | 11.02 | M |  | 9.622 |  |
| AGS1060 | 11.25 | M |  | 12.559 |  |
| AGS1060 | 11.31 | M |  | 8.861 |  |
| AGS1119 | 3.17 | M | AGS | 12.116 | c.428G>A p.Arg143His hom |
| AGS1119 | 3.28 | M |  | 9.908 |  |
| AGS1119 | 3.69 | M |  | 17.404 |  |
| AGS1119 | 3.70 | M |  | 16.399 |  |
| AGS1289 | 0.77 | M | AGS | 6.041 | Ex13_14del hom |
| AGS1289 | 0.79 | M |  | 4.876 |  |
| AGS1460 | 4.81 | F | AGS | 6.567 | c.602T>A p.Ile201Asn het; c.1293A>T p.Leu431Phe het |
| AGS1572 | 0.22 | F | AGS | 8.142 | c.625G>A p.Gly209Ser het + c.1343T>C p.Ile448Thr het |

M male; F female; het heterozygous; hom homozygous; AGS Aicardi-Goutières syndrome; SP spastic paraparesis; FCL familial chilblain lupus. Nucleotide numbering based on transcript *SAMHD1* NM_015474.3. Protein numbering based on transcript NP_056289.2. ^*SAMHD1* c.1609-1G>C hom + *ADAR1* c.1684G>A p.Ala562Thr het §*SAMHD1* c.1693G>A p.Ala565Thr hom + *RNASEH2B* c.529G>A p.Ala177Thr het.

**Table S8** Age, sex, interferon score and genotype for 34 patients (28 families, 56 measurements) with mutations in *ADAR1*

| **Patient number** | **Age at sampling (Years)** | **Sex** | **Phenotype** | **Interferon score** | **Mutation(s)** |
| --- | --- | --- | --- | --- | --- |
| AGS081_P1 | 14.53 | F | AGS | 24.267 | c.577 C>G p.Pro193Ala het; c.2675 G>A p.Arg892His het |
| AGS081_P1 | 15.01 | F |  | 53.356 |  |
| AGS081_P1 | 15.78 | F |  | 45.676 |  |
| AGS081_P2 | 4.82 | M | AGS | 37.822 | c.577 C>G p.Pro193Ala het; c.2675 G>A p.Arg892His het |
| AGS081_P2 | 5.28 | M |  | 21.590 |  |
| AGS093 | 15.26 | M | AGS | 25.608 | c.577 C>G p.Pro193Ala het; c.2608 G>A p. Ala870Thr het |
| AGS093 | 16.59 | M |  | 46.665 |  |
| AGS107 | 15.26 | F | AGS | 64.220 | c.3337 G>C p.Asp1113His hom |
| AGS150 | 10.88 | F | AGS | 14.690 | c.3019 G>A p.Gly1007Arg het *de novo* |
| AGS251 | 8.1 | F | BSN | 28.367 | c.577 C>G p.Pro193Ala het; c.2615 T>C p.Ile872Thr het |
| AGS251 | 9.27 | F |  | 12.301 |  |
| AGS327_P1 | 4.07 | M | AGS/BSN/DSH | 23.382 | c.577 C>G p.Pro193Ala het; c.1076_1080del p.Lys359Argfs*14 het |
| AGS327_P2 | 0.03 | F | Clinically unaffected | 7.301 | c.577 C>G p.Pro193Ala het; c.1076_1080del p.Lys359Argfs*14 het |
| AGS327_P2 | 0.26 | F |  | 6.547 |  |
| AGS327_P2 | 0.61 | F |  | 1.737 |  |
| AGS327_P2 | 1.09 | F |  | 4.720 |  |
| AGS430_P1 | 4.75 | M | AGS | 8.296 | c.577 C>G p.Pro193Ala het; c.2675 G>A p.Arg892His het |
| AGS430_P1 | 5.53 | M |  | 21.538 |  |
| AGS430_P2 | 4.75 | M | AGS | 12.444 | c.577 C>G p.Pro193Ala het; c.2675 G>A p.Arg892His het |
| AGS430_P2 | 5.53 | M |  | 14.306 |  |
| AGS474 | 5.42 | M | AGS | 20.961 | c.3019 G>A p.Gly1007Arg het *de novo* |
| AGS474 | 5.88 | M |  | 32.319 |  |
| AGS474 | 6.02 | M |  | 49.463 |  |
| AGS530_P1 | 26.21 | F | BSN | 23.385 | c.3019 G>A p.Gly1007Arg het† |
| AGS530_P2 | 13.41 | F | Dystonia | 12.502 | c.3019 G>A p.Gly1007Arg het† |
| AGS550 | 8.39 | M | AGS | 6.429 | c.577 C>G p.Pro193Ala het; c.2565_2568 del p.Asn857Alafs*17 het |
| AGS567 | 2.81 | M | BSN | 36.387 | c.518 A>G p.Asn173Ser het; c.2515del p.Thr839Profs*6 het |
| AGS663 | 17.53 | M | BSN | 39.488 | c.577 C>G p.Pro193Ala het; c.1630 C>T p.Arg544* het |
| AGS679 | 1.66 | F | BSN | 3.802 | c.577C>G p.Pro193Ala het; c.3556A>G p.Lys1186Glu het |
| AGS683 | 8.29 | M | SP | 23.187 | c.3365A>T p.Lys1122Met hom |
| AGS683 | 8.71 | M |  | 15.483 |  |
| AGS699 | 4.91 | M | SP | 16.833 | c.3019G>A p.Gly1007Arg het Inheritance not determined |
| AGS703 | 8.44 | M | BSN | 20.427 | c.577C>G p.Pro193Ala het; c.3100A>G p.Met1034Val het |
| AGS703 | 8.44 | M |  | 29.817 |  |
| AGS720 | 6.90 | F | BSN | 12.057 | c.577C>G p.Pro193Ala het; c.2250del p.Gly751Aspfs*42 het |
| AGS759 | 4.09 | F | BSN/hemiplegia | 11.048 | c.577C>G p.Pro193Ala het; c.2902G>A p.Asp968Asn |
| AGS759 | 4.53 | F |  | 18.633 |  |
| AGS788 | 1.29 | F | BSN | 1.99 | c.577C>G p.Pro193Ala het; c.1383_1387delTGCT p.Asp462Glufs*2 het |
| AGS788 | 2.46 | F |  | 4.596 |  |
| AGS810_P1 | 7.13 | M | AGS | 40.571 | c.3019 G>A p.Gly1007Arg het |
| AGS810_P1 | 7.27 | M |  | 14.851 |  |
| AGS810_P2 | 33.34 | F | IBGC | 25.743 | c.3019 G>A p.Gly1007Arg het |
| AGS810_P2 | 33.48 | F |  | 12.836 |  |
| AGS943 | 11.75 | M | SP | 24.753 | c.3019G>A p.Gly1007Arg het *de novo* |
| AGS943 | 12.11 | M |  | 15.074 |  |
| AGS1014 | 9.66 | F | Neurocognitive impairment | 1.449 | c.577C>G p.Pro193Ala hom |
| AGS1170 | 0.84 | F | BSN | 17.627 | c.577 C>G p.Pro193Ala het; c.3100A>G Met1034Val het |
| AGS1170 | 0.90 | F |  | 1.158 |  |
| AGS1170 | 1.23 | F |  | 3.578 |  |
| AGS1315_P1 | 3.06 | M | SP+ICC | 17.147 | c.3019G>A p.Gly1007Arg het^ |
| AGS1315_P2 | 5.53 | M | SP+ICC | 10.506 | c.3019G>A p.Gly1007Arg het^ |
| AGS1456 | 16.68 | M | AGS-like | 9.063 | c.577C>G p.Pro193Ala het; c.3020-3C>G het |
| AGS1507 | 8.56 | M | BSN/DSH | 8.293 | c.2763-2A>G het; c.577C>G p.Pro193Ala het |
| AGS1542 | 6.38 | M | SP | 12.24 | c.3335A>T p.Tyr1112Phe hom |
| AGS1542 | 6.43 | M |  | 18.051 |  |
| AGS1824 | 5.00 | M | BSN | 8.713 | c.577C>G p.Pro193Ala het; c.1084_1085delAG p.Arg362Aspfs*12 het |

M male; F female; het heterozygous; hom homozygous; AGS Aicardi-Goutières syndrome; SP spastic paraparesis; BSN bilateral striatal necrosis; DSH Dyschromatosis Symmetrica Hereditaria; ICC intracranial calcification; IBGC idiopathic basal ganglia calcification; † paternally inherited. Nucleotide numbering based on transcript *ADAR1* NM_001111.4. Protein numbering based on transcript NP_001102.2. ^Inherited from Father who is mosaic for mutation. Values in green are below the 2.466 cut-off.

**Table S9** Age, sex, interferon score and genotype for 26 patients (18 families, 59 measurements) with mutations in *IFIH1*

| **Patient number** | **Age at sampling (Years)** | **Sex** | **Phenotype** | **Interferon score** | **Mutation(s)** |
| --- | --- | --- | --- | --- | --- |
| AGS237 | 12.55 | M | AGS | 12.263 | c.1009A>G p.Arg337Gly het *de novo* |
| AGS237 | 12.60 | M |  | 22.699 |  |
| AGS259_P1 | 8.14 | M | AGS | 19.223 | c.2336G>A p.Arg779His het |
| AGS259_P1 | 8.21 | M |  | 35.919 |  |
| AGS259_P1 | 8.29 | M |  | 26.725 |  |
| AGS259_P2 | 48.80 | M | Clinically unaffected | 10.828 | c.2336G>A p.Arg779His het |
| AGS259_P2 | 48.87 | M |  | 16.999 |  |
| AGS259_P2 | 48.95 | M |  | 29.076 |  |
| AGS259_P2 | 49.73 | M |  | 20.348 |  |
| AGS259_P3 | 79.33 | F | Clinically unaffected | 12.278 | c.2336G>A p.Arg779His het |
| AGS259_P3 | 79.41 | F |  | 7.017 |  |
| AGS259_P3 | 80.19 | F |  | 19.378 |  |
| AGS524_P1 | 3.91 | F | AGS | 20.085 | c.1483 G>A p.Gly495Arg het |
| AGS524_P1 | 5.19 | F |  | 25.448 |  |
| AGS524_P1 | 5.83 | F |  | 41.012 |  |
| AGS524_P1 | 7.36 | F |  | 13.14 |  |
| AGS524_P2 | 32.34 | M | SP | 15.167 | c.1483 G>A p.Gly495Arg het *de novo* |
| AGS524_P2 | 32.85 | M |  | 11.512 |  |
| AGS524_P2 | 33.13 | M |  | 17.853 |  |
| AGS524_P2 | 33.62 | M |  | 29.342 |  |
| AGS524_P2 | 33.95 | M |  | 9.900 |  |
| AGS626 | 17.14 | M | AGS | 12.364 | c.1178A>T p.Asp393Val het *de novo* |
| AGS626 | 17.66 | M |  | 15.626 |  |
| AGS647 | 1.39 | M | AGS | 22.033 | c.2159G>A p.Arg720Gln het *de novo* |
| AGS647 | 1.96 | M |  | 16.116 |  |
| AGS647 | 2.06 | M |  | 18.412 |  |
| AGS674 | 9.44 | M | AGS | 21.774 | c.992C>G p.Thr331Arg het *de novo* |
| AGS723 | 15.20 | F | ICC | 39.454 | c.2335C>T p.Arg779Cys het |
| AGS735 | 14.57 | M | AGS | 18.858 | c.2471G>A p.Arg824Lys het *de novo* |
| AGS735 | 14.75 | M |  | 20.101 |  |
| AGS755_P1 | 2.16 | M | FCL | 7.537 | c.1465G>A p.Ala489Thr het |
| AGS755_P1 | 2.47 | M |  | 21.328 |  |
| AGS755_P1 | 3.04 | M |  | 59.766 |  |
| AGS755_P2 | 3.82 | M | FCL | 7.291 | c.1465G>A p.Ala489Thr het |
| AGS755_P2 | 4.70 | M |  | 31.919 |  |
| AGS755_P3 | 41.00 | M | FCL | 9.845 | c.1465G>A p.Ala489Thr het |
| AGS755_P3 | 41.88 | M |  | 16.721 |  |
| AGS807 | 33.08 | M | SMS | 10.715 | c.2465G>A p.Arg822Gln het |
| AGS826_P1 | 48.75 | M | SMS | 6.39 | c.2465G>A p.Arg822Gln het |
| AGS826_P2 | 59.97 | M | SMS | 5.02 | c.2465G>A p.Arg822Gln het |
| AGS829 | 17.75 | F | SMS | 26.076 | c.2465G>A p.Arg822Gln het *de novo* |
| AGS848_P1 | 3.52 | M | SP | 20.844 | c.1165G>A p.Gly389Arg het§ |
| AGS848_P1 | 4.30 | M |  | 12.495 |  |
| AGS848_P1 | 4.30 | M |  | 23.086 |  |
| AGS848_P2 | 37.88 | M | SP | 23.7 | c.1165G>A p.Gly389Arg het |
| AGS848_P2 | 38.44 | M |  | 11.953 |  |
| AGS848_P2 | 38.66 | M |  | 5.901 |  |
| AGS848_P2 | 38.66 | M |  | 9.549 |  |
| AGS848_P2 | 39.63 | M |  | 9.859 |  |
| AGS848_P3 | 78.67 | F | Clinically unaffected | 11.96 | c.1165G>A p.Gly389Arg het |
| AGS848_P3 | 78.89 | F |  | 11.145 |  |
| AGS848_P3 | 78.89 | F |  | 13.909 |  |
| AGS1001 | 14.72 | M | AGS | 24.47 | c.1347C>G p.Asn449Lys het *de novo* |
| AGS1004 | 4.97 | M | AGS | 20.212 | c.2335C>T p.Arg779Cys het *de novo* |
| AGS1156 | 1.66 | M | AGS | 12.545 | c.2335C>T p.Arg779Cys het *de novo* |
| AGS1156 | 1.81 | M |  | 12.776 |  |
| AGS1351 | 1.47 | F | AGS | 12.494 | c.2336G>A p.Arg779His het *de novo* |
| AGS1351 | 1.81 | F |  | 10.431 |  |
| AGS1514 | 6.32 | M | AGS/SMS | 7.229 | c.2465G>A p.Arg822Gln het |

M male; F female; het heterozygous; hom homozygous; AGS Aicardi-Goutières syndrome; SP spastic paraparesis; SMS Singleton-Merton syndrome; FCL familial chilblain lupus; ICC intracranial calcification. Nucleotide numbering based on transcript *IFIH1* NM_022168.3. Protein numbering based on transcript NP_071451. §*IFIH1* c.1165G>A p.Gly389Arg het + *RNASEH2B* c.529G>A pAla177Thr het.

**Table S10** Age, sex, interferon score and genotype for 12 patients (10 families, 17 measurements) with mutations in *ACP5*

| **Patient number** | **Age at sampling (Years)** | **Sex** | **Phenotype** | **Interferon score** | **Mutation(s)** |
| --- | --- | --- | --- | --- | --- |
| AGS170 | 26 | F | SPENCD | 49.393 | g.11,543,542-11,558,411 del p.Ex4_7 del hom |
| AGS170 | 27 | F |  | 40.034 |  |
| AGS241 | 7.54 | M | SPENCD | 13.931 | c.369C>A p.Tyr123* het; c.721G>A p.sp241Asn het |
| AGS242_P1 | 12.44 | M | SPENCD | 26.168 | c.266C>T p.Thr89Ile hom |
| AGS242_P1 | 12.51 | M |  | 21.388 |  |
| AGS242_P1 | 16.50 | M |  | 14.826 |  |
| AGS242_P2 | 15.35 | F | SPENCD | 3.757 | c.266C>T p.Thr89Ile hom |
| AGS242_P2 | 19.33 | F |  | 30.404 |  |
| AGS262 | 11 | M | SPENCD | 71.094 | c.667C>T p.Gln223* hom |
| AGS402 | 15.96 | F | SPENCD | 34.120 | c.643G>A p.Gly215Arg hom |
| AGS631_P1 | 12.66 | F | SPENCD | 24.840 | c.155A>C p.Lys52Thr hom; c.790A>G p.Met264Val het |
| AGS631_P2 | 10.82 | M | SPENCD | 24.816 | c.155A>C p.Lys52Thr hom; c.790A>G p.Met264Val het |
| AGS657 | 36.36 | F | SPENCD | 2.77 | c.325G>A p.Gly109Arg hom |
| AGS724 | 9.39 | M | SPENCD | 0.726 | c.359A>G p.Gln120Arg hom |
| AGS724 | 9.52 | M |  | 0.6 |  |
| AGS775 | 41.46 | M | SPENCD | 0.770 | c.325G>A p.Gly109Arg hom |
| AGS1161 | 28.24 | M | SPENCD | 21.427 | c.335A>T p.Asp112Val hom |

M male; F female; het heterozygous; hom homozygous; SPENCD Spondyloenchondrodysplasia. Nucleotide numbering based on transcript *Acp5* NM_001111035.1. Protein numbering based on transcript NP_001104505. Values in green are below the 2.466 cut-off.

**Table S11** Age, sex, interferon score and genotype for 10 patients (8 families, 14 measurements) with mutations in *TMEM173*

| **Patient number** | **Age at sampling (Years)** | **Sex** | **Phenotype** | **Interferon score** | **Mutation(s)** |
| --- | --- | --- | --- | --- | --- |
| AGS831_P1 | 3.76 | F | SAVI | 63.73 | c.463G>A p.Val155Met het |
| AGS831_P2 | 33.90 | M | SAVI | 39.428 | c.463G>A p.Val155Met het |
| AGS831_P3 | 65.31 | M | SAVI | 8.693 | c.463G>A p.Val155Met het |
| AGS876 | 8.00 | M | SAVI | 20.566 | c.439G>A p.Val147Met het |
| AGS964 | 9.46 | F | SAVI | 22.831 | c.463G>A p.Val155Met het |
| AGS1058 | 6.50 | F | SAVI | 12.341 | c.842g>a p.Arg281Gln het |
| AGS1058 | 6.83 | F |  | 6.208 |  |
| AGS1058 | 7.00 | F |  | 13.963 |  |
| AGS1182 | 11.27 | M | SAVI | 18.141 | c.463G>A p.Val155Met het |
| AGS1295 | 13.90 | M | SAVI | 27.535 | c.463G>A p.Val155Met het |
| AGS1295 | 14.21 | M |  | 25.76 |  |
| AGS1429 | 7.49 | M | SAVI | 11.522 | c.461A>G p.Asn154Ser het |
| AGS1429 | 7.69 | M |  | 9.823 |  |
| AGS1802 | 16.12 | M | SAVI | 6.561 | c.617G>A p.Cys206Tyr het |

M male; F female; het heterozygous; hom homozygous; SAVI Stimulator of Interferon Genes-Associated Vasculopathy with Onset in Infancy. Nucleotide numbering based on transcript *TMEM173* NM_198282.3. Protein numbering based on transcript NP_938023.

**Table S12** Age, sex, interferon score and genotype for 4 patients (4 families, 5 measurements) with mutations in complement genes

| **Patient number** | **Age at sampling (Years)** | **Sex** | **Phenotype** | **Interferon score** | **Gene and Mutation(s)** |
| --- | --- | --- | --- | --- | --- |
| AGS412 | 3 | F | Complement deficiency | 44.230 | *C1qB* c.287del G p.Gly96Alafs*50 hom |
| AGS846 | 17.84 | F | Complement deficiency | 11.904 | *C2* c.839_849+17delTGGTGGACAGGGTCAGGAATCAGGAGTC hom |
| AGS1000 | 15.13 | F | Complement deficiency | 8.795 | *C1qA* c.208C>T p.Gln208* hom |
| AGS1000 | 16.01 | F |  | 2.9 |  |
| AGS1614 | 2.77 | F | Complement deficiency | 24.934 | *C1qA* c.79C>T p.Arg27* hom |

M male; F female; het heterozygous; hom homozygous. Nucleotide numbering based on transcript *C1qA* NM_015991.2. Protein numbering based on transcript C1qA NP_057075. Nucleotide numbering based on transcript *C1qB* NM_000491.3. Protein numbering based on transcript C1qB NP_000482. Nucleotide numbering based on transcript *C2* NM_000063.4. Protein numbering based on transcript C2 NP_000054.

**Table S13** Age, sex, interferon score and genotype for 4 patients (3 families, 5 measurements) with mutations in *ISG15*

| **Patient number** | **Age at sampling (Years)** | **Sex** | **Phenotype** | **Interferon score** | **Mutation(s)** |
| --- | --- | --- | --- | --- | --- |
| AGS1213 | 13 | F | IBGC | 18.245 | c.163C>T p.Gln 55* hom |
| AGS1214 | 11 | F | IBGC | 25.288 | c.163C>T p.Gln 55* hom |
| AGS1217 | 14 | M | MSMD | 21.240 | c.339dup p.Leu114Alafs*? hom |
| AGS1217 | 16 | M |  | 8.189 |  |
| AGS1218 | 16 | F | MSMD | 38.257 | c.379G>T p.Glu127* hom |

M male; F female; het heterozygous; hom homozygous; IBGC idiopathic basal ganglia calcification; MSMD Mendelian susceptibility to mycobacterial disease. Nucleotide numbering based on transcript *ISG15* NM_005101.3 Protein numbering based on transcript NP_005092.

**Table S14** Age, sex, interferon score and genotype for 2 patients (2 families, 2 measurements) with mutations in *SKIV2L*

| **Patient number** | **Age at sampling (Years)** | **Sex** | **Phenotype** | **Interferon score** | **Mutation(s)** |
| --- | --- | --- | --- | --- | --- |
| AGS1233 | 1 | M | THES | 22.039 | c.3561_3581del p.Ser1189_Leu1195del hom |
| AGS1234 | 4 | F | THES | 27.310 | c.848G>A p.Trp283* het; c.1022T>G p.Val341Gly het |

M male; F female; het heterozygous; hom homozygous; THES trichohepatoenteric syndrome. Nucleotide numbering based on transcript *SKIV2L* NM_006929.4. Protein numbering based on transcript NP_008860.

**Table S15** Age, sex, interferon score and genotype for 2 patients (2 families, 3 measurements) with mutations in *DNASE1L3*

| **Patient number** | **Age at sampling (Years)** | **Sex** | **Phenotype** | **Interferon score** | **Mutation(s)** |
| --- | --- | --- | --- | --- | --- |
| AGS501 | 4.86 | F | JSLE | 11.003 | c.290_291delCA p.Thr97Ilefs*2 het; Ex5 del het |
| AGS501 | 9.02 | F |  | 0.374 |  |
| AGS919 | 12.18 | M | JSLE | 10.825 | c.290_291delCA p.Thr97Ilefs*2 hom |

M male; F female; het heterozygous; hom homozygous; JSLE juvenile systemic lupus erythematosus. Nucleotide numbering based on transcript *DNASE1L3* NM_004944.3. Protein numbering based on transcript NP_004935. Value in green is below the 2.466 cut-off.

**Table S16** Age, sex, interferon score and genotype for 2 patients (2 families, 6 measurements) with mutations in *PRKDC*

| **Patient number** | **Age at sampling (Years)** | **Sex** | **Phenotype** | **Interferon score** | **Mutation(s)** |
| --- | --- | --- | --- | --- | --- |
| AGS639 | 11.56 | M | Autoimmunity, immunodeficiency, granuloma | 26.513 | c.9185T>G p.Leu3062Arg hom |
| AGS639 | 13.31 | M |  | 1.312 |  |
| AGS639 | 13.87 | M |  | 19.182 |  |
| AGS639 | 14.29 | M |  | 5.672 |  |
| AGS705 | 20.15 | F | Autoimmunity, immunodeficiency, granuloma | 20.887 | c.9185T>G p.Leu3062Arg hom |
| AGS705 | 22.01 | F |  | 14.934 |  |

M male; F female; het heterozygous; hom homozygous. Nucleotide numbering based on transcript *PRKDC* NM_006904.6. Protein numbering based on transcript NP_008835. Value in green is below the 2.466 cut-off.

**Table S17** Age, sex, interferon score and genotype for 5 patients (3 families, 11 measurements) with mutations in *CECR1*

| **Patient number** | **Age at sampling (Years)** | **Phenotype** | **Sex** | **Interferon score** | **Mutation(s)** |
| --- | --- | --- | --- | --- | --- |
| AGS750 | 6.73 | Inflammatory | M | 5.154 | c.139G>C p.Gly47Arg hom |
| AGS750 | 6.81 |  | M | 7.053 |  |
| AGS750 | 7.02 |  | M | 3.349 |  |
| AGS750 | 7.56 |  | M | 1.295 |  |
| AGS785_P1 | 37.19 | Inflammatory | F | 5.989 | c.578C>T p.Pro193Leu het |
| AGS785_P2 | 22.48 | Inflammatory | M | 5.783 | c.506G>A p.Arg169Gln het; c.578C>T p.Pro193Leu het |
| AGS785_P2 | 22.56 |  | M | 5.006 |  |
| AGS975_P1 | 8.88 | Inflammatory | F | 5.653 | c.1358A>G p.Tyr453Cys; c.973-?_1081+?del p.Val325Thrfs*7 |
| AGS975_P1 | 9.03 |  | F | 1.86 |  |
| AGS975_P2 | 7.77 | Inflammatory | F | 11.877 | c.1358A>G p.Tyr453Cys; c.973-?_1081+?del p.Val325Thrfs*7 |
| AGS975_P2 | 7.92 |  | F | 9.465 |  |

M male; F female; het heterozygous; hom homozygous. Nucleotide numbering based on transcript *CECR1* NM_001282225.1. Protein numbering based on transcript NP_059120. Values in green are below the 2.466 cut-off.

**Table S18** Age, sex, interferon score and genotype for 5 patients (4 families, 6 measurements) with mutations in *RNASET2*

| **Patient number** | **Age at sampling (Years)** | **Phenotype** | **Sex** | **Interferon score** | **Mutation(s)** |
| --- | --- | --- | --- | --- | --- |
| AGS077 | 20 | Cystic leukoencephalopathy without megalencephaly | M | 1.492 | c.550T>C p.Cys184Arg hom |
| AGS774 | 2.03 | Cystic leukoencephalopathy without megalencephaly | M | 7.761 | c.397_399del p.Lys133del het; c.145G>T p.Glu49* het |
| AGS1048_P1 | 10.88 | Cystic leukoencephalopathy without megalencephaly | F | 2.852 | c.2del p.0? hom |
| AGS1048_P2 | 2.69 | Cystic leukoencephalopathy without megalencephaly | F | 5.864 | c.2del p.0? hom |
| AGS1197 | 4.34 | Cystic leukoencephalopathy without megalencephaly | M | 4.356 | c.202T>C p.Trp68Arg het; c.710dupG p.Leu238Serfs*5 het |
| AGS1197 | 4.34 |  | M | 4.219 |  |

M male; F female; het heterozygous; hom homozygous. Nucleotide numbering based on transcript *RNASET2* NM_003730.4. Protein numbering based on transcript NP_003721. Value in green is below the 2.466 cut-off.

**Table S19** Age, sex, interferon score and genotype for 3 patients (3 families, 4 measurements) with mutations in *TRNT1*

| **Patient number** | **Age at sampling (Years)** | **Sex** | **Phenotype** | **Interferon score** | **Mutation(s)** |
| --- | --- | --- | --- | --- | --- |
| AGS980 | 3.56 | F | SIFD | 8.053 | c.1057-7C>G het + c.1213G>A p.Gly450Arg het |
| AGS980 | 3.78 | F |  | 1.283 |  |
| AGS1089 | 8.41 | M | SIFD | 1.448 |  |
| AGS1819 | 23.00 | M | SIFD | 6.647 | c.295C>T p.Arg99Trp hom |

M male; F female; het heterozygous; hom homozygous; SIFD Sideroblastic anemia with B-cell immunodeficiency, periodic fevers and developmental delay. Nucleotide numbering based on transcript *TRNT1* NM_182916.2. Protein numbering based on transcript NP_886552. Values in green are below the 2.466 cut-off.

**Table S20** Age, sex, and interferon score for 55 patients (50 families, 78 measurements) with juvenile systemic lupus erythematosus (JSLE)

| **Patient number** | **Age at sampling (Years)** | **Sex** | **Interferon score** | **Detailed clinical phenotype** | **Treatment** |
| --- | --- | --- | --- | --- | --- |
| AGS463_P1 | 17.19 | F | 14.925 | JSLE (onset 4 years old) | NA |
| AGS463_P1 | 18.31 | F | 23.538 |  | NA |
| AGS463_P1 | 19.20 | F | 23.386 |  | NA |
| AGS463_P1 | 20.40 | F | 1.611 |  | Thalidomide |
| AGS463_P2 | 54.52 | F | 25.989 | SLE | None |
| AGS463_P3 | 32.20 | F | 4.011 | SLE + polymyositis (overlap syndrome) | NA |
| AGS571 | 7.03 | F | 14.416 | SLE with cerebral vasculopathy (onset ?) | NA |
| AGS700 | 16.26 | M | 14.969 | Kikuchi | NA |
| AGS700 | 16.59 | M | 7.008 |  | NA |
| AGS700 | 16.69 | M | 0.735 |  | NA |
| AGS832 | 15.25 | F | 13.086 | JSLE (onset 2 years old), failure to thrive, severe skin disease, ILD | Steroids, hydroxychloroquine |
| AGS832 | 15.92 | F | 32.782 |  | Steroids, hydroxychloroquine |
| AGS833 | 11.66 | M | 18.475 | JSLE (onset 4 years old), failure to thrive, chilblain lupus, pyramidal syndrome, no calcifications, chronic inflammatory syndrome | Hydroxychloroquine |
| AGS833 | 12.37 | M | 11.957 |  | Steroids, hydroxychloroquine |
| AGS836_P1 | 6.98 | M | 5.725 | JSLE, lupus nephritis, bronchiectasis | Steroids, hydroxychloroquine, mycophenolate mofetil |
| AGS836_P2 | 14.08 | F | 0.349 |  | Steroids, hydroxychloroquine, mycophenolate mofetil |
| AGS836_P2 | 14.17 | F | 0.99 |  | Steroids, hydroxychloroquine, mycophenolate mofetil |
| AGS871 | 12.97 | F | 32.918 | JSLE (onset 4 years old), thrombosis disease (spleen infarction, uvula necrosis), severe neutrophilic urticarial lesion. | Rituximab, hydroxychloroquine |
| AGS871 | 13.09 | F | 23.931 |  | Rituximab, hydroxychloroquine |
| AGS871 | 14.24 | F | 21.693 |  | Anakinra (anti-IL1), hydroxychloroquine |
| AGS871 | 14.68 | F | 35.483 |  | None |
| AGS873 | 14.20 | F | 0.82 | JSLE (onset 14 years old), lupus nephritis, pleuritis, hematological involvement | Cyclophosphamide, pulses of steroids, hydroxychloroquine |
| AGS873 | 14.83 | F | 16.256 |  | Steroids, hydroxychloroquine |
| AGS885 | 14.63 | F | 5.364 | JSLE (onset 13 years old), cutaneo-articular lupus and thrombocytopenia | Steroids, azathioprine |
| AGS898 | 11.69 | F | 11.706 | JSLE (onset 12 years old), cutaneo-articular lupus, hepatitis | Steroids, azathioprine |
| AGS898 | 12.09 | F | 11.307 |  | Steroids, azathioprine |
| AGS930 | 49.43 | F | 12.602 | JSLE |  |
| AGS937 | 4.59 | F | 110.705 | Severe JSLE, more STING-like, bronchiectasis. | Steroids, cyclophosphamide |
| AGS937 | 4.59 | F | 96.292 |  | Steroids |
| AGS939 | 19.62 | M | 16.662 | JSLE (onset 14 years old) with pulmonary involvement, angioedema, intra-alveolar haemorrhage. | Rituximab, hydroxychloroquine, aspirin |
| AGS939 | 19.96 | M | 19.451 |  | Steroids, hydroxychloroquine |
| AGS949 | 18.93 | F | 9.047 | SLE. Myalgia, arthralgia/transient arthritis, malar rash, cytopenia. Bone marrow transplant 4 years previously for chronic granulomatous (mutations in *p47PHOX*). | Steroids |
| AGS995 | 11.80 | F | 14.912 | JSLE (onset 12 years old), Kikuchi (10 years old) | None |
| AGS995 | 12.11 | F | 6.284 |  | None |
| AGS995 | 12.70 | F | 26.95 |  | None |
| AGS995 | 13.32 | F | 5.494 |  | Hydroxychloroquine |
| AGS1017 | 14.93 | M | 0.929 | JSLE with neurolupus. Haemophagocitic syndrome post EBV activation. | Pulse of steroids |
| AGS1017 | 15.08 | M | 1.906 |  | Steroids, cyclophosphamide |
| AGS1054 | 10.53 | F | 24.079 | JSLE (onset 8 years old following Toxocara canis infection) with cutaneous vasculitis | Steroids, hydroxychloroquine |
| AGS1068 | 7.76 | F | 3.039 | JSLE (onset ?) with vasculopathy | Steroids, hydroxychloroquine |
| AGS1074 | 15.52 | M | 1064.16 | JSLE (onset 14 years old) with intracranial hypertension. Kikuchi | None |
| AGS1074 | 16.24 | M | 7.058 |  | None |
| AGS1074 | 16.47 | M | 17.439 |  | None |
| AGS1078 | 14.29 | F | 11.393 | JSLE (onset 14 years old) with anti factor XIII antibody and neutrophilic dermatitis | Steroids, rituximab |
| AGS1078 | 14.97 | F | 7.478 |  | Steroids, hydroxychloroquine, colchicine |
| AGS1079 | 16.43 | F | 6.102 | JSLE (onset 15 years old) with hepatitis, pancreatitis, MAS and retinal vasculitis. Kikuchi | Steroids, hydroxychloroquine |
| AGS1123 | 8.90 | F | 0.402 | JSLE (onset 6 years old) | Methotrexate, hydroxychloroquine |
| AGS1124 | 11.90 | M | 0.650 | Cutaneous lupus (onset 7 years old) | NA |
| AGS1128_P1 | 25.50 | F | 4.609 | JSLE | NA |
| AGS1128_P2 | 27.29 | M | 0.557 | JSLE | NA |
| AGS1129 | 16.72 | F | 12.817 | JSLE | NA |
| AGS1130 | 50.12 | F | 5.568 | JSLE | NA |
| AGS1131_P1 | 52.90 | F | 0.176 | JSLE | NA |
| AGS1131_P2 | 20.38 | F | 0.95 | JSLE | NA |
| AGS1132 |  |  | 9.837 | JSLE | NA |
| AGS1133 |  |  | 14.912 | JSLE | NA |
| AGS1159 | 15.74 | F | 22.508 | JSLE (onset 15 years old). Sickle cell disease | Steroids, hydroxychloroquine, mycophenolate mofetil |
| AGS1196 | 12.93 | F | 16.462 | JSLE (onset 12 years old) with severe lupus nephritis. Familial lupus | None |
| AGS1203 | 15.27 | M | 2.763 | JSLE (onset 9 years old) with cerebral vasculopathy and short stature | Steroids, hydroxychloroquine, mycophenolate mofetil |
| AGS1248 | 14.22 | F | 1.87 | JSLE (onset 12 years old) and obesity | Mycophenolate mofetil |
| AGS1265 | 14.70 | F | 8.075 | JSLE (onset 14 years old) with autoinflammation | Steroids, mycophenolate mofetil |
| AGS1330 | 12.94 | M | 9.891 | JSLE (onset 12 years old) with lupus nephritis. Familial lupus | Steroids, hydroxychloroquine, mycophenolate mofetil |
| AGS1355 | 11.04 |  | 16.837 | JSLE | NA |
| AGS1364 | 15.79 |  | 6.242 | JSLE SLEDAI NA | NA |
| AGS1366 | 11.75 |  | 1.046 | JSLE SLEDAI = 0 | NA |
| AGS1371 | 16.11 |  | 3.911 | JSLE SLEDAI = 2 | NA |
| AGS1372 | 15.51 |  | 16.244 | JSLE SLEDAI = 1 | NA |
| AGS1377 | 18.58 |  | 22.374 | JSLE SLEDAI = 9 | NA |
| AGS1431 | 10.41 | F | 17.207 | JSLE (onset 9 years old) with autoimmune hepatitis | None |
| AGS1451 | 12.13 | F | 6.572 | JSLE (onset 12 years old) with severe lupus nephritis | Pulse of steroids, cyclophosphamide |
| AGS1462 | 10.49 | F | 2.661 | JSLE with neurolupus (onset 8 years old) | None |
| AGS1465 | 20.08 | F | 5.767 | JSLE (onset 15 years old). Kikuchi | Hydroxychloroquine |
| AGS1550 | 14.72 | F | 7.423 | JSLE (onset 13 years old) with atypical phenotype. Carpal destructive synovitis, chilblain lupus | None |
| AGS1550 | 15.20 | F | 8.328 |  | NSAIDS |
| AGS1577 | 12.02 | F | 20.468 | JSLE (onset 12 years old) | Steroids, hydroxychloroquine |
| AGS1589 | 6.46 | M | 13.209 | JSLE (onset 6 years old) with interstitial and alveolar lung disease | None |
| AGS1599 | 17.21 | F | 11.75 | JSLE (onset 4 years old) with lupus nephritis, severe skin vasculitis, chilblains. Kidney graft at 5 years old | NA |
| AGS1765 | 9.94 | F | 3.556 | JSLE (onset ?) with neurolupus and APLS | Steroids, hydroxychloroquine, mycophenolate mofetil, aspirin |

Values in green are below the 2.466 cut-off. JSLE juvenile systemic lupus erythematosus; ILD interstitial lung disease. SLEDAI, SLE disease activity index; APLS antiphospholipid syndrome; MAS macrophage activation syndrome; NSAIDS non-steroidal anti-inflammatory drugs; NA not available. All cases conform to American College of Rheumatology criteria for a diagnosis of SLE.

**Table S21** Age, sex and interferon score for 59 patients (59 families, 101 measurements) with juvenile dermatomyositis (JDM) / overlap myositis (OM)

| **Patient number** | **Age at sampling (Years)** | **Sex** | **Interferon score** | **Detailed clinical phenotype** | **Treatment** |
| --- | --- | --- | --- | --- | --- |
| AGS857 | 4.26 | M | 25.915 | JDM (onset 3 years old) with severe bowel vasculitis. Anti-Tiff1 g antibodies | Steroids, mycophenolate mofetil |
| AGS857 | 5.04 | M | 18.974 |  | Steroids, mycophenolate mofetil, hydroxychloroquine |
| AGS857 | 5.47 | M | 11.158 |  | Steroids, nivaquine, immunoadsorption |
| AGS857 | 6.18 | M | 20.271 |  | Steroids, hydroxychloroquine |
| AGS857 | 6.21 | M | 18.766 |  | Steroids, rituximab, IgIV |
| AGS859 | 6.56 | F | 16.741 | JDM (onset 7 years old) | Steroids |
| AGS875 | 19.50 | F | 23.668 | JDM (onset 6 years old) and neurological syndrome (pontocerebella hyperplasia). Calcinosis. Anti-Tiff1 g antibodies | Steroids |
| AGS900 | 8.42 | F | 30.902 | JDM (onset 5 years old). No myositis specific antibodies | Steroids, mycophenolate mofetil |
| AGS900 | 8.56 | F | 1.178 |  | Steroids, mycophenolate mofetil, IgIV |
| AGS900 | 9.63 | F | 30.185 |  | Steroids, mycophenolate mofetil |
| AGS907 | 11.16 | F | 20.066 | JDM (onset 11 years old). Anti-NXP2 antibodies | Steroids, methotrexate |
| AGS907 | 11.66 | F | 2.386 |  | Steroids, methotrexate |
| AGS918 | 13.67 | F | 8.451 | OM (onset 8 years old). Anti-MDA5 antibodies | Steroids |
| AGS918 | 14.34 | F | 26.195 |  | Steroids |
| AGS938 | 12.81 | F | 9.202 | OM (onset 8 years old), with discoid lupus. Severe calcinosis. Aseptic abscesses. anti-U1 RNP antibodies | Steroids, mycophenolate mofetil |
| AGS940 | 5.88 | F | 46.68 | JDM (onset 4 years old). | Methotrexate |
| AGS941 | 3.68 | F | 19.822 | JDM (onset 3 years old). Calcinosis. Anti-NXP2 antibodies | None |
| AGS942 | 10.98 | F | 6.256 | JDM (onset 3 years old) with bowel vasculitis. Calcinosis. No myositis specific antibodies | Mycophenolate mofetil |
| AGS945 | 9.10 | M | 26.752 | Very severe JDM (onset 8 years old). Capillary leak syndrome. Failure to thrive. Anti-Tiff1 g antibodies | Pulse of steroids, methotrexate, hydroxychloroquine |
| AGS945 | 10.08 | M | 4.639 |  | Steroids, rituximab, IgIV, hydroxychloroquine |
| AGS945 | 10.27 | M | 56.063 |  | Steroids, rituximab, hydroxychloroquine, immunoadsorption |
| AGS946 | 8.18 | F | 2.737 | JDM (onset 6 years old) with ILD and bowel vasculitis. Calcinosis. Anti-NXP2 and anti-factor H antibodies | Steroids, IgIV, hydroxychoroquine |
| AGS947 | 14.89 | F | 16.117 | JDM (onset 14 years old). Anti-NXP2 antibodies | Pulse of steroids |
| AGS947 | 14.95 | F | 8.277 |  | Steroids, methotrexate, IgIV |
| AGS948 | 15.06 | M | 37.089 | OM (onset 3 years old) with sclerodermia and renal involvement. Pulmonary fibrosis. Anti-MDA5 antibodies. | Azathioprine, hydroxychloroquine |
| AGS948 | 15.45 | M | 31.795 |  | Mycophenolate mofetil, hydroxychloroquine |
| AGS948 | 16.35 | M | 13.66 |  | Mycophenolate mofetil, hydroxychloroquine |
| AGS960 | 10.55 | M | 0.379 | JDM (onset 5 years old). Calcinosis. No myositis specific antibodies | Steroids, methotrexate, NSAIDS |
| AGS960 | 10.84 | M | 2.628 |  | Steroids, cyclosporin, NSAIDS |
| AGS976 | 13.17 | M | 0.419 | JDM (onset 7 years old) with lipodystrophy and poikilodermia. No myositis specific antibodies | Steroids, mycophenolate mofetil |
| AGS984 | 11.67 | M | 5.041 | OM (onset 2 years old) | NA |
| AGS1011 | 11.94 | F | 17.747 | OM (onset 9 years old) with lupus features and failure to thrive. Anti-U1 RNP, anti-MDA5 and anti-SmB antibodies | Steroids, mycophenolate mofetil, hydroxychloroquine, aspirin |
| AGS1011 | 12.79 | F | 24.58 |  | Steroids, mycophenolate mofetil, hydroxychloroquine, mesalazine |
| AGS1022 | 13.20 | F | 18.553 | OM (onset 9 years old) with lupus features and insulin-dependent diabetes mellitus. Anti-U1 RNP antibodies | None |
| AGS1050 | 3.89 | M | 0.211 | JDM (onset 2 years old). Calcinosis. Anti-NXP2, anti-PM/Scl75, anti-SRP antibodies | Steroids, methotrexate |
| AGS1050 | 4.14 | M | 0.592 |  | Steroids, methotrexate |
| AGS1050 | 4.49 | M | 0.466 |  | Mycophenolate mofetil, methotrexate |
| AGS1051 | 12.96 | F | 13.783 | JDM (onset 11 years old) with bowel vasculitis. Anti-NXP2 antibodies | Pulse of steroids |
| AGS1051 | 13.08 | F | 17.778 |  | Steroids, methotrexate |
| AGS1051 | 13.20 | F | 27.121 |  | Steroids, methotrexate |
| AGS1066 | 7.62 | M | 27.615 | JDM (onset 2 years old). No myositis specific antibodies | None |
| AGS1067 | 10.51 | F | 4.086 | JDM (onset 9 years old) with ILD. No myositis specific antibodies | None |
| AGS1067 | 10.91 | F | 0.146 |  | Steroids, methotrexate |
| AGS1073 | 12.47 | F | 10.201 | Severe JDM (onset 10 years old) with severe bowel vasculitis, hepatitis, diabetes and capillary leak syndrome. Anti-NXP2 antibodies | Hydrocortisone, plasmapheresis |
| AGS1073 | 12.90 | F | 6.846 |  | Hydrocortisone, plasmapheresis |
| AGS1073 | 13.14 | F | 5.586 |  | Hydrocortisone, hydroxychloroquine |
| AGS1073 | 13.35 | F | 3.916 |  | Hydrocortisone, rituximab |
| AGS1091 | 14.47 | M | 11.974 | JDM (onset 11 years old). Diffuse calcinosis. Anti-MDA5 and anti-PL12 antibodies | Methotrexate |
| AGS1093 | 11.21 | M | 29.808 | JDM (onset 10 years old). Anti-MDA5 antibodies | Steroids, methotrexate |
| AGS1093 | 11.57 | M | 2.159 |  | Steroids, methotrexate, hydroxychloroquine |
| AGS1093 | 11.88 | M | 0.707 |  | Steroids, methotrexate, hydroxychloroquine |
| AGS1103 | 10.77 | F | 3.078 | JDM (onset 10 years old) with failure to thrive. Calcinosis | Methotrexate |
| AGS1103 | 11.10 | F | 3.018 |  | Steroids, methotrexate |
| AGS1105 | 13.34 | F | 31.846 | JDM (onset 13 years old) with bowel vasculitis. Anti-NXP2 antibodies | Pulse of steroids, methotrexate |
| AGS1105 | 14.06 | F | 21.494 |  | Steroids, rituximab |
| AGS1106 | 12.02 | F | 18.311 | JDM (onset 11 years old) with ILD. Anti-MDA5 antibodies | None |
| AGS1113 | 16.20 | F | 0.849 | OM (onset 16 years old) with macrophage activation syndrome and mild renal involvement. Anti-U1 RNP, anti-SSA antibodies | Steroids |
| AGS1114 | 12.03 | F | 8.541 | OM (onset 12 years old). Anti-U1 RNP, anti-SSA antibodies | Steroids, methotrexate |
| AGS1124 | 14.49 | F | 0.58 | JDM (onset 3 years old) with a brother with lupus-like disease | None |
| AGS1126 | 15.29 | M | 0.491 | JDM (onset ?). No myositis specific autoantibodies | Steroids, methotrexate |
| AGS1162 | 16.99 | F | 8.958 | JDM (onset 7 years old). Calcinosis. No myositis specific autoantibodies | Steroids, methotrexate, hydroxychloroquine, mycophenolate mofetil |
| AGS1175 | 13.72 | F | 43.057 | JDM (onset 13 years old). No myositis specific autoantibodies | Steroids |
| AGS1175 | 14.21 | F | 0.256 |  | Steroids, methotrexate |
| AGS1177 | 12.85 | F | 9.872 | JDM (onset 10 years old). Anti-Tiff1 g antibodies | Steroids, methotrexate, hydroxychloroquine |
| AGS1193 | 8.65 | M | 8.492 | JDM (onset 8 years old). Anti-Tiff1 g antibodies | None |
| AGS1193 | 9.17 | M | 4.205 |  | Steroids, methotrexate |
| AGS1194 | 12.40 | F | 0.823 | OM (onset 10 years old) with localized scleroderma, ILD and eosinophilic colitis. Anti-PM/Scl antibodies | NA |
| AGS1245 | 17.87 | M | 8.298 | JDM (onset 12 years old). Antibodies NA | Methotrexate, hydroxychloroquinine |
| AGS1247 | 7.80 | M | 5.486 | JDM (onset 7 years old). Calcinosis. Anti-Mi2alpha antibodies | NA |
| AGS1269 | 16.22 | M | 0.386 | JDM (onset?). Severe calcinosis. Anti-Tiff1 g antibodies | Steroids, mycophenolate mofetil |
| AGS1269 | 17.13 | M | 58.375 |  | NA |
| AGS1274 | 8.38 | F | 9.521 | OM (onset 8 years old) with lupic features. Anti-beta2 Gp1 and no myositis specific antibodies | Steroids, methotrexate, hydroxychloroquine |
| AGS1274 | 8.99 | F | 14.433 |  | Steroids, hydroxychloroquine |
| AGS1302 | 11.72 | F | 10.98 | Very severe OM (onset 10 years old). Anti-Tiff1 g antibodies | Methotrexate, mycophenolate mofetil |
| AGS1302 | 12.50 | F | 4.014 |  | NA |
| AGS1302 | 12.55 | F | 6.689 |  | Eculizumab, mycophenolate mofetil |
| AGS1302 | 12.60 | F | 4.872 |  | NA |
| AGS1302 | 12.64 | F | 9.019 |  | NA |
| AGS1302 | 12.68 | F | 25.205 |  | Eculizumab, plasmapheresis |
| AGS1302 | 12.81 | F | 18.124 |  | Eculizumab, plasmapheresis |
| AGS1302 | 12.85 | F | 8.296 |  | Eculizumab, plasmapheresis |
| AGS1305 | 9.08 | F | 6.389 | JDM (onset 3 years old). Calcinosis. Anti-NXP2 antibodies | None |
| AGS1320 | 11.47 | M | 12.261 | JDM (onset 11 years old). Anti-NXP2 antibodies | Pulse of steroids, methotrexate, hydroxychloroquine |
| AGS1320 | 12.24 | M | 0.966 |  | Steroids, methotrexate, hydroxychloroquine |
| AGS1326 | 14.78 | F | 24.734 | JDM (onset 14 years old) with ILD and bowel vasculitis. Anti-NXP2 antibodies | Steroids |
| AGS1326 | 15.31 | F | 0.131 |  | Steroids, methotrexate, hydroxychloroquine |
| AGS1326 | 15.56 | F | 0.644 |  | Steroids, methotrexate, hydroxychloroquine |
| AGS1469 | 29.16 | M | 1.119 | JDM (childhood onset). Antibodies NA | None |
| AGS1511 | 9.33 | F | 21.961 | JDM (onset 4 years old) with bowel vasculitis. Anti-NXP2 antibodies | None |
| AGS1512 | 44.01 | F | 4.078 | OM (adult onset) with lung fibrosis. Anti-PL12 antibodies | None |
| AGS1527 | 16.11 | F | 9.306 | OM (onset 11 years old) with ILD. Anti-cardiolipin and anti-U1 RNP antibodies | None |
| AGS1528 | 3.86 | F | 2.273 | OM (onset 4 years old). Anti-fibrillarin antibodies | None |
| AGS1554 | 13.18 | F | 27.862 | Very severe JDM (onset 13 years old). Anti-NXP2 antibodies | Steroids, methotrexate |
| AGS1582 | 19.00 | F | 27.469 | Atypical OM (onset 2 years old) with ICC, MAS and cutaneous vasculitis with chilblains. Anti-U1 RNP and anti-SM antibodies | Steroids, methotrexate, hydroxychloroquine |
| AGS1601 | 13.30 | F | 0.902 | OM (onset 12 years old) with features of lupus and scleroderma. Anti-SSA, anti-Ku and anti-fibrillarin antibodies | Steroids, methotrexate, hydroxychloroquine |
| AGS1601 | 13.37 | F | 64.756 |  | Steroids, methotrexate, hydroxychloroquine |
| AGS1612 | 6.86 | F | 0.658 | Amyopathic JDM (onset 4 years old). No myositis specific antibodies | Hydroxychloroquine |
| AGS1725 | 14.73 | M | 0.558 | JDM (onset 8 years old). Calcinosis. No myositis specific antibodies | Mycophenolate mofetil |
| AGS1732 | 9.21 | M | 22.439 | JDM (onset 9 years old). No myositis specific antibodies | None |
| AGS1753 | 6.92 | F | 35.918 | JDM (onset 6 years old). No myositis specific antibodies | None |
| AGS1756 | 6.30 | M | 0.43 | JDM (onset 3 years old). No myositis specific antibodies | None |

Values in green are below the 2.466 cut-off. JDM juvenile dermatomyositis (according to criteria of Troyanov)^1^. OM Overlap myositis (according to criteria of Troyanov)^1^; ILD interstitial lung disease; ICC intracranial calcification; MAS macrophage activation syndrome; NA not available.

**Table S22** Age, sex, phenotype and interferon score for 24 patients (17 families, 72 measurements) with a type 1 interferonopathy phenotype negative for mutations in *AGS1-7*

| **Patient number** | **Age at sampling (Years)** | **Sex** | **Phenotype** | **Interferon score** |
| --- | --- | --- | --- | --- |
| AGS271 | 9.86 | F | AGS-like | 8.758 |
| AGS271 | 10.16 | F |  | 8.714 |
| AGS271 | 10.61 | F |  | 11.459 |
| AGS271 | 11.62 | F |  | 8.552 |
| AGS406_P1 | 12.49 | M | FCL-like | 7.664 |
| AGS406_P1 | 13.52 | M |  | 11.295 |
| AGS406_P1 | 14.27 | M |  | 8.108 |
| AGS406_P2 | 38.93 | F | FCL-like | 12.101 |
| AGS406_P2 | 40.71 | F |  | 11.50 |
| AGS406_P3 | 7.35 | F | FCL-like | 14.158 |
| AGS406_P3 | 7.58 | F |  | 15.92 |
| AGS406_P3 | 8.39 | F |  | 8.401 |
| AGS406_P3 | 9.13 | F |  | 6.779 |
| AGS458 | 3.10 | M | AGS-like | 4.681 |
| AGS458 | 3.55 | M |  | 1.901 |
| AGS458 | 4.70 | M |  | 28.346 |
| AGS472 | 12.37 | F | SPENCD-like | 6.346 |
| AGS472 | 14.45 | F |  | 108.360 |
| AGS472 | 14.95 | F |  | 64.070 |
| AGS520_P1 | 4.48 | F | AGS-like | 9.801 |
| AGS520_P1 | 4.83 | F |  | 7.269 |
| AGS520_P2 | 8.69 | M |  | 22.420 |
| AGS520_P2 | 9.04 | M |  | 7.398 |
| AGS520_P3 | 1.73 | M |  | 9.912 |
| AGS520_P3 | 2.08 | M |  | 10.114 |
| AGS556 | 2.55 | F | AGS | 9.995 |
| AGS556 | 2.65 | F |  | 12.329 |
| AGS556 | 3.13 | F |  | 13.06 |
| AGS556 | 5.40 | F |  | 14.341 |
| AGS561 | 17.48 | M | AGS-like | 8.404 |
| AGS561 | 18.53 | M |  | 4.792 |
| AGS561 | 18.74 | M |  | 5.879 |
| AGS611_P1 | 48.20 | F | FCL-like | 0.690 |
| AGS611_P1 | 48.40 | F |  | 0.914 |
| AGS611_P2 | 15.73 | F | FCL-like | 7.219 |
| AGS611_P2 | 15.94 | F |  | 7.865 |
| AGS670 | 5.31 | F | SPENCD-like | 39.098 |
| AGS670 | 6.21 | F |  | 23.560 |
| AGS670 | 7.89 | F |  | 16.855 |
| AGS694_P1 | 2.57 | F | SPENCD-like | 10.245 |
| AGS694_P1 | 2.81 | F |  | 11.549 |
| AGS694_P1 | 4.60 | F |  | 10.604 |
| AGS694_P2 | 7.77 | F | SPENCD-like | 36.482 |
| AGS695 | 1.75 | M | AGS-like | 8.834 |
| AGS695 | 2.06 | M |  | 6.347 |
| AGS695 | 2.67 | M |  | 7.878 |
| AGS797 | 12.42 | F | Skin + neurology | 15.995 |
| AGS797 | 12.68 | F |  | 6.243 |
| AGS797 | 12.95 | F |  | 15.584 |
| AGS797 | 13.31 | F |  | 10.61 |
| AGS797 | 14.24 | F |  | 8.267 |
| AGS851_P1 | 5.98 | M | Chronic meningeal inflammation | 3.203 |
| AGS851_P1 | 6.40 | M |  | 37.581 |
| AGS851_P1 | 7.46 | M |  | 83.777 |
| AGS851_P1 | 7.62 | M |  | 4.58 |
| AGS851_P1 | 7.79 | M |  | 2.441 |
| AGS851_P2 | 8.38 | F | Chronic meningeal inflammation | 1.747 |
| AGS851_P2 | 9.28 | F |  | 52.646 |
| AGS909 | 8.49 | F | AGS-like | 13.362 |
| AGS909 | 9.01 | F |  | 8.969 |
| AGS909 | 10.28 | F |  | 10.746 |
| AGS936 | 14.00 | M | Dystonia | 5.294 |
| AGS936 | 14.92 | M |  | 4.667 |
| AGS936 | 15.17 | M |  | 5.285 |
| AGS1339 | 2.22 | M | STING-like | 8.527 |
| AGS1339 | 2.50 | M |  | 68.829 |
| AGS1339 | 2.61 | M |  | 36.261 |
| AGS1574 | 0.78 | M | AGS-like | 4.941 |
| AGS1574 | 0.88 | M |  | 8.705 |
| AGS1574 | 0.93 | M |  | 10.328 |
| AGS1574 | 1.23 | M |  | 13.452 |
| AGS1574 | 1.33 | M |  | 6.64 |

Values in green are below the 2.466 cut-off. AGS Aicardi-Goutières syndrome; FCL familial chilblain lupus; SPENCD Spondyloenchondrodysplasia

**Table S23** Age, sex and interferon score for 41 patients (37 families, 58 measurements) with a non-molecularly confirmed clinical autoinflammatory phenotype

| **Patient number** | **Age at sampling (Years)** | **Sex** | **Detailed clinical phenotype** | **Interferon score** | **Treatment** |
| --- | --- | --- | --- | --- | --- |
| AGS583 | ?16 | M | Congenital autoinflammatory | 1.156 | Infliximab, methotrexate |
| AGS665 | 5.07 | F | Recurrent fever, joint pain, fatigue, sore throat | 0.639 | Colchicine |
| AGS665 | 5.16 | F |  | 7.416 | Colchicine. Cured by tonsillectomy |
| AGS668 | 14.80 | M | Recurrent fever, malaise, rash | 3.650 | Colchicine |
| AGS668 | 14.91 | M |  | 0.342 | Colchicine |
| AGS668 | 15.17 | M |  | 0.302 | Colchicine |
| AGS668 | 15.32 | M |  | 0.489 | Colchicine |
| AGS692 | 4.68 | F | Beçhet-like | 3.569 | Steroids |
| AGS692 | 4.98 | F | Beçhet-like | 1.270 | Steroids |
| AGS692 | 5.00 | F | Beçhet-like | 41.932 | Steroids |
| AGS714 | 1.26 | F | NA | 1.127 | Hydroxychloroquine |
| AGS744 | 6.45 | M | Recurrent fever, bowel urgency, joint pain, malaise | 0.315 | Colchicine |
| AGS744 | 6.72 | M |  | 2.209 | Colchicine |
| AGS790_P1 | 36.19 | F | SPA. Vascular disease, polyarthritis | 0.959 | None |
| AGS790_P2 | 1.90 | F | Recurrent fever. Vascular disease. Cerebral stroke | 1.047 | None |
| AGS790_P2 | 2.19 | F |  | 0.775 | None |
| AGS791 | 3.90 | F | Recurrent fevers, cytophagic panniculitis, lipoatrophy, lupic biology | 4.000 | Hydroxychloroquine, steroids |
| AGS791 | 4.58 | F |  | 0.878 | mycophenolate mofetil |
| AGS818 | 10.20 | M | Recurrent fevers, cytophagic panniculitis, mild lipoatrophy, joint pain and chilblains. Failure to thrive | 32.12 | None |
| AGS818 | 10.37 | M |  | 9.652 | Pulse of steroids |
| AGS818 | 10.70 | M |  | 18.7 | Steroids, cyclosporine |
| AGS818 | 11.68 | M |  | 6.783 | Steroids, cyclosporine |
| AGS818 | 12.29 | M |  | 19.309 | Steroids, cyclosporine |
| AGS858 | 14.98 | M | Recurrent fever, adenitis | 1.243 | None |
| AGS877 | 2.49 | M | Recurrent fevers, joint pain, urticarial rush | 0.11 | Steroids, anakinra (anti IL1) |
| AGS908 | 7.45 | M | GI and skin vasculitis (polyarteritis nodosa-like) | 0.203 | None |
| AGS908 | 7.82 | M |  | 0.734 | Steroids, azathioprine |
| AGS982 | 9.69 | F | Recurrent fevers, joint pain, enthesitis | 0.485 | Colchicine |
| AGS990 | 13.03 | M | Recurrent pericarditis and pleuritis, post-surgical Dressler syndrome | 0.624 | Aspirin, colchicine |
| AGS1012 | 7.13 | M | Recurrent ileitis, joint pain. Henoch-Schönlein purpura. | 0.488 | Steroids |
| AGS1026 | 12.40 | M | Recurrent fevers, hypergammaglobulinemia, splenomegaly, neutrophilic meningoencephalitis with white matter lesions and CNS vasculitis | 1.494 | Colchicine |
| AGS1039 | 4.66 | M | Recurrent fevers with regressive neutropenia. Delay in speech | 0.979 | None |
| AGS1041 | 6.32 | F | Recurrent fevers. Lobular panniculitis. Hypogammaglobulinemia | 4.519 | IV Ig |
| AGS1092 | 9.91 | F | Recurrent fevers. Pyramidal syndrome | 3.322 | Colchicine |
| AGS1092 | 10.31 | F |  | 3.092 | Colchicine |
| AGS1121 | 3.92 | M | Recurrent fevers. Panniculitis with vasculitis. Cyclic neutropenia. Eosinophilic ileitis and colitis | 1.261 | Dapsone-pyrimethamine |
| AGS1157 | 5.32 | M | Recurrent fevers. Vasculitis, cytopenia | 0.277 | NA |
| AGS1168 | 8.47 | M | Recurrent fevers, hepatosplenomegaly, joint pain, hypergammaglobulinemia, anaemia. Sensorineural hearing loss | 0.417 | Colchicine |
| AGS1176 | 3.11 | M | Recurrent fever. Panniculitis with lipoatrophy | 0.726 | NA |
| AGS1202 | 49.08 | F | FMF with M694I homozygous mutation in MEVF | 3.111 | Tociluzimab (anti IL6) |
| AGS1297_P1 | 30.18 | F | Arthritis, recurrent fevers, cutaneous vasculitis-like lesions | 2.151 | Anakinra (anti IL1) |
| AGS1297_P2 | 0.13 | M | Neonatal onset recurrent fever, failure to thrive, hepatosplenomegalia, chronic dyspnea | 0.245 | Steroids |
| AGS1338 | 0.14 | M | Recurrent fevers, lipoatrophy, inflammatory bowel disease, developmental delay, immunodeficiency | 5.456 | Anakinra (anti IL1) |
| AGS1338 | 0.27 | M |  | 3.823 | Anakinra (anti IL1) |
| AGS1443_P1 | 1.80 | M | Recurrent fevers with aphtosis, pharyngitis and adenopathies, rash | 8.15 | None |
| AGS1443_P2 | 0.46 | F | Recurrent fevers with aphtosis, pharyngitis and adenopathies, rash | 2.043 | None |
| AGS1506 | 1.51 | F | Recurrent fevers, urticarial rash, hypereosinophilia | 4.254 | Canakinumab (anti IL1) |
| AGS1506 | 1.64 | F |  | 7.303 | Anakinra (anti IL1) |
| AGS1553 | 7.59 | M | Recurrent fevers, epidermolysis bullosa simplex | 3.255 | Colchicine |
| AGS1576 | 14.45 | F | Recurrent pericarditis | 0.339 | None |
| AGS1598 | 14.05 | F | Behçet-like disease, thrombogenic features | 0.378 | None |
| AGS1606 | 6.88 | M | Recurrent fevers with pericarditis and pleuritis. Fibroblastic rheumatism. Raynaud’s syndrome | 0.41 | Colchicine |
| AGS1720_P1 | 5.40 | F | Recurrent fevers with aseptic purulent meningitis. Spastic paraparesis and developmental delay. White matter lesions. | 15.984 | Anakinra (anti IL1) |
| AGS1720_P2 | 9.97 | M | Recurrent fevers with aseptic purulent meningitis. Spastic paraparesis and developmental delay. White matter lesions | 0.918 | None |
| AGS1751 | 10.95 | F | Recurrent fevers, HIGD-like | 1.921 | NA |
| AGS1760 | 4.58 | M | Recurrent skin vasculitis, Henoch-Schönlein purpura-like | 1.129 | None |
| AGS1836 | 1.73 | F | Recurrent fevers, urticarial rash, hypereosinophilia | 6.717 | Canakinumab (anti IL1) |
| AGS1943 | 4.65 | F | Recurrent fevers. Desquamatization hands. Arthritis | 1.113 | Methotrexate |

Values in green are below the 2.466 cut-off. GI gastro-intestinal; CNS central nervous system; SPA spondylarthopathy; HIGD hyper Ig D syndrome; FMF familial Mediterranean fever; NA not available.

**Table S24** Age, sex and interferon score for 19 patients (17 families, 21 measurements) with a systemic juvenile arthritis (sJIA) phenotype

| **Patient number** | **Age at sampling (Years)** | **Sex** | **Clinical detailed phenotype** | **Interferon score** | **Treatment** |
| --- | --- | --- | --- | --- | --- |
| AGS872 | 15.16 | M | sJIA with ANCA nephritis | 45.851 | Canakinumab (anti-IL1) |
| AGS872 | 15.85 | M |  | 0.333 | Canakinumab (anti-IL1) |
| AGS1037 | 8.12 | F | sJIA with pericarditis | 0.345 | Anakinra (anti-IL1) |
| AGS1038_P1 | 7.96 | F | sJIA | 0.477 | Colchicine |
| AGS1038_P2 | 10.72 | F | sJIA | 2.269 | Colchicine |
| AGS1038_P3 | 1.28 | F | sJIA | 0.873 | NA |
| AGS1040 | 9.31 | M | sJIA with MAS, acanthosis nigricans, lipodystrophy | 0.866 | Steroids, anakinra (anti-IL1) |
| AGS1096 | 10.55 | M | sJIA | 0.628 | Steroids |
| AGS1200 | 16.33 | F | sJIA, autoimmune hepatitis, ANCA+, anti-DNA+ | 0.676 | Abatacept, azathioprine, steroids |
| AGS1243 | 19.39 | M | sJIA | 0.595 | Canakinumab (anti-IL1) |
| AGS1267 | 28.68 | F | sJIA with ANCA nephritis. Kidney transplant | 0.483 | Tacrolimus, steroids |
| AGS1270 | 10.85 | F | sJIA | 1.217 | NA |
| AGS1273 | 7.16 | M | sJIA | 0.498 | Steroids, anakinra (anti-IL1) |
| AGS1333 | 15.50 | F | sJIA | 0.544 | Canakinumab (anti-IL1) |
| AGS1472 | 14.85 | F | sJIA | 16.587 | Canakinumab (anti-IL1) |
| AGS1472 | 15.02 | F |  | 8.138 | Canakinumab (anti-IL1) |
| AGS1609 | 8.84 | F | sJIA | 11.258 | Canakinumab (anti-IL1) |
| AGS1724 | 14.11 | M | sJIA | 4.704 | Anakinra (anti-IL1) |
| AGS1738 | 9.55 | M | sJIA | 0.493 | Steroids, methotrexate, canakinumab (anti-IL1) |
| AGS1764 | 12.08 | M | sJIA | 0.62 | Tociluzimab (anti-IL6) |
| AGS1851 | 14.31 | F | sJIA | 21.699 | Anakinra (anti-IL1) |

Values in green are below the 2.466 cut-off. sJIA; systemic juvenile idiopathic arthritis; MAS macrophage activation syndrome.

**Table S25** Age, sex and interferon score for 9 patients (7 families, 10 measurements) with a non-systemic juvenile polyarthritis (pJIA) phenotype

| **Patient number** | **Age at sampling (Years)** | **Sex** | **Detailed clinical phenotype** | **Interferon score** | **Treatment** |
| --- | --- | --- | --- | --- | --- |
| AGS620_P1 | 6.56 | M | Familial pJIA with systemic inflammation (no criteria of sJIA) | 0.405 | NA |
| AGS620_P2 | 20.42 | F | Familial pJIA with systemic inflammation (no criteria of sJIA) | 0.415 | Methotrexate |
| AGS620_P3 | 13.98 | F | Familial pJIA with systemic inflammation (no criteria of sJIA) | 0.981 | NA |
| AGS620_P3 | 14.10 | F | Familial pJIA with systemic inflammation (no criteria of sJIA) | 1.088 | NA |
| AGS1024 | 4.90 | F | pJIA with systemic inflammation (no criteria of sJIA) | 1.044 | NSAIDS |
| AGS1463 | 8.25 | F | Mono JIA | 1.391 | None |
| AGS1516 | 9.64 | F | Oligo JIA | 0.332 | None |
| AGS1591 | 11.71 | F | Oligo JIA | 0.618 | NA |
| AGS1835 | 4.99 | M | Arthritis | 1.069 | Steroids, methotrexate |
| AGS1848 | 4.21 | F | Oligo JIA (SLE in mother) | 0.271 | None |

Values in green are below the 2.466 cut-off. pJIA; non-systemic juvenile polyarthritis.

**Table S26** Age, sex, interferon score and genotype for group 1 patients with a negative score on at least one occasion

| **Patient number** | **Age at sampling (Years)** | **Sex** | **Phenotype** | **Interferon score** | **Genes and Mutation(s)** |
| --- | --- | --- | --- | --- | --- |
| AGS525 | 3.5 | F | Mild neurodevelopmental delay | 1.542 | *TREX1* c.152_153del p.Gln51Argfs*50 het; c.197 A>G p.Lys66Arg het |
| AGS525 | 3.5 | F |  | 2.439 |  |
| AGS1116 | 6.14 | F | FCL | 3.946 | *TREX1* c.52G>A p.Asp18Asn het |
| AGS1116 | 6.19 | F |  | 5.889 |  |
| AGS1116 | 6.23 | F |  | 0.568 |  |
| AGS015 | 12.01 | M | AGS | 2.149 | *RNASEH2B* c.510+1 G>A het; c.529 G>A p.Ala177Thr het |
| AGS026_P1 | 20.35 | M | AGS | 1.668 | *RNASEH2B* c.136+1 del het; c.529 G>A p.Ala177Thr het |
| AGS026_P1 | 20.64 | M |  | 4.495 |  |
| AGS026_P1 | 20.71 | M |  | 4.401 |  |
| AGS049 | 11.59 | M | AGS | 0.699 | *RNASEH2B* c.529 G>A p.Ala177Thr hom |
| AGS067 | 8.24 | F | AGS | 1.050 | *RNASEH2B* c.529 G>A p.Ala177Thr het; c.128 C>A p.Pro43His het |
| AGS144 | 13.26 | F | AGS | 1.48 | *RNASEH2B* c.529G>A p.Ala177Thr hom |
| AGS144 | 13.34 | F |  | 0.63 |  |
| AGS144 | 13.49 | F |  | 0.927 |  |
| AGS220 | 6.4 | F | AGS | 0.718 | *RNASEH2B* c.136+1 del het; c.529 G>A p.Ala177Thr het |
| AGS230_P1 | 13.78 | M | AGS | 0.656 | *RNASEH2B* c.529G>A p.Ala177Thr hom |
| AGS230_P2 | 15.67 | M | AGS | 0.568 | *RNASEH2B* c.529G>A p.Ala177Thr hom |
| AGS231 | 9.25 | F | AGS | 2.355 | *RNASEH2B* c.529G>A p.Ala177Thr het; c.634G>A p.Ala212Thr het |
| AGS297 | 7.99 | M | AGS | 2.374 | *RNASEH2B* c.529G>A p.Ala177Thr hom |
| AGS301 | 6.26 | M | AGS | 2.329 | *RNASEH2B* c.529G>A p.Ala177Thr hom |
| AGS301 | 6.37 | M |  | 1.518 |  |
| AGS304 | 10.37 | F | AGS | 1.558 | *RNASEH2B* c.488C>T p.Thr163Ile het; c.529G>A p.Ala177Thr het |
| AGS486 | 2.36 | F | AGS | 13.198 | *RNASEH2B* c.529G>A p.Ala177Thr hom |
| AGS486 | 4.03 | F |  | 2.437 |  |
| AGS539 | 3.35 | M | AGS | 1.635 | *RNASEH2B* c.529G>A p.Ala177Thr hom |
| AGS539 | 3.66 | M |  | 2.638 |  |
| AGS558 | 3.14 | F | AGS | 1.299 | *RNASEH2B* c.529G>A p.Ala177Thr hom |
| AGS673 | 4.63 | M | AGS | 2.769 | *RNASEH2B* c.529G>A p.Ala177Thr hom |
| AGS673 | 4.99 | M |  | 1.709 |  |
| AGS688 | 1.63 | M | AGS | 5.424 | *RNASEH2B* c.529G>A p.Ala177Thr hom |
| AGS688 | 2.02 | M |  | 2.398 |  |
| AGS707 | 8.81 | M | AGS | 25.741 | *RNASEH2B* c.529G>A p.Ala177Thr hom |
| AGS707 | 8.94 | M |  | 1.583 |  |
| AGS711 | 2.82 | F | SP | 4.031 | *RNASEH2B* c.529G>A p.Ala177Thr hom |
| AGS711 | 3.31 | F |  | 1.312 |  |
| AGS739_P2 | 10.66 | F | SP | 1.337 | *RNASEH2B* c.529G>A p.Ala177Thr hom |
| AGS760 | 6.69 | F | AGS | 2.065 | *RNASEH2B* c.488C>T p.Thr163Ile; c.844A>T p.Lys295Ile |
| AGS768 | 5.69 | M | SP | 1.900 | *RNASEH2B* c.529G>A p.Ala177Thr hom |
| AGS793 | 11.26 | M | AGS | 0.242 | *RNASEH2B* c.529G>A p.Ala177Thr hom |
| AGS825 | 4.84 | F | AGS | 0.640 | *RNASEH2B* c.529G>A p.Ala177Thr hom |
| AGS825 | 4.91 | F |  | 0.960 |  |
| AGS834 | 6.39 | F | AGS | 0.709 | *RNASEH2B* c.529G>A p.Ala177Thr het; c.412C>T p.Leu138Phe het |
| AGS1097_P2 | 4.88 | M | AGS | 0.451 | *RNASEH2B* c.529G>A p.Ala177Thr hom |
| AGS1097_P2 | 5.95 | M |  | 54.887 |  |
| AGS1122 | 15.45 | M | AGS | 2.418 | *RNASEH2B* c.529G>A p.Ala177Thr het; c.511-2A>G het |
| AGS1522_P1 | 12.57 | F | AGS | 1.644 | *RNASEH2B* c.529G>A p.Ala177Thr hom |
| AGS1525 | 4.60 | M | AGS | 2.018 | *RNASEH2B* c.529G>A p.Ala177Thr hom |
| AGS327_P2 | 0.03 | F | Clinically unaffected | 7.301 | *ADAR1* c.577 C>G p.Pro193Ala het; c.1076_1080del p.Lys359Argfs*14 het |
| AGS327_P2 | 0.26 | F |  | 6.547 |  |
| AGS327_P2 | 0.61 | F |  | 1.737 |  |
| AGS327_P2 | 1.09 | F |  | 4.720 |  |
| AGS788 | 1.29 | F | AGS | 1.99 | *ADAR1* c.577C>G p.Pro193Ala het; c.1383_1387delTGCT p.Asp462Glufs*2 het |
| AGS788 | 2.46 | F |  | 4.596 |  |
| AGS1014 | 9.66 | F | neurocognitive impairment | 1.449 | *ADAR1* c.577C>G p.Pro193Ala hom |
| AGS1170 | 0.84 | F | AGS/BSN | 17.627 | *ADAR1* c.577 C>G p.Pro193Ala het; c.3100A>G Met1034Val het |
| AGS1170 | 0.90 | F |  | 1.158 |  |
| AGS1170 | 1.23 | F |  | 3.578 |  |
| AGS724 | 9.39 | M | SPENCD | 0.726 | *ACP5* c.359A>G p.Gln120Arg hom |
| AGS724 | 9.52 | M |  | 0.6 |  |
| AGS775 | 41.46 | M | SPENCD | 0.770 | *ACP5* c.325G>A p.Gly109Arg hom |

M male; F female; het heterozygous; hom homozygous; AGS Aicardi-Goutières syndrome; FCL familial chilblain lupus; BSN bilateral striatal necrosis; SP spastic paraparesis; SPENCD Spondyloenchondrodysplasia. Pale blue TREX1, pink RNASEH2B, brown ADAR1, bright blue ACP5.

**Supplementary Reference**

1. Troyanov Y, Targoff IN, Tremblay JL, Goulet JR, Raymond Y, Senecal JL. Novel classification of idiopathic inflammatory myopathies based on overlap syndrome features and autoantibodies: analysis of 100 French Canadian patients. *Medicine (Baltimore).* 2005;84(4):231-249.

**Figure S1** Interferon score of controls with more than one measurement over time

Data shown are interferon scores plotted against time since first sampling (years). Interferon scores calculated from the median fold change in RQ values for a panel of six interferon stimulated genes (ISGs). Blue dashed line represents boundary of positive score (> 2.466). Number of serial samples for each control shown in brackets in legend.

**Figure S2** Relative quantification of each interferon stimulated gene in group 1 patients plotted by genotype

Interferon stimulated gene (ISG) RQ (relative quantification) by genotype for 267 mutation positive group 1 patients (458 measurements) compared with controls. Horizontal black bars show the median RQ value for each probe in each genotype. Analysed with one-way ANOVA with Dunnett’s multiple comparison test. RQ is equal to 2^-∆∆Ct^ i.e. the normalized fold change relative to a control.

**Figure S3** Relative quantification of each interferon stimulated gene in group 2 patients plotted by genotype

Interferon stimulated gene (ISG) RQ (relative quantification) by genotype for 17 mutation positive group 2 patients (30 measurements) compared with controls. Horizontal black bars show the median RQ value for each probe in each genotype. Analysed with one-way ANOVA with Dunnett’s multiple comparison test. RQ is equal to 2^-∆∆Ct^ i.e. the normalized fold change relative to a control.

**Figure S4** Relative quantification of each interferon stimulated gene in group 3 patients plotted by genotype

Interferon stimulated gene (ISG) RQ (relative quantification) by genotype for 207 group 3 patients (340 measurements) compared with controls. Horizontal black bars show the median RQ value for each probe in each genotype. Analysed with one-way ANOVA with Dunnett’s multiple comparison test. RQ is equal to 2^-∆∆Ct^ i.e. the normalized fold change relative to a control.

**Figure S5** Interferon score for parents and siblings of group 1 patients

Interferon score plotted for each parental sample according to genotype. Black horizontal lines represent median for each genotype. Interferon scores calculated from the median fold change in RQ (relative quantification) values for a panel of six interferon stimulated genes (ISGs). Blue dots represent an interferon score less than 2.466. Red dots represent an interferon score greater than 2.466. Analysed by one-way ANOVA with Dunnett’s multiple comparison test.
